# DJ-1 is a redox sensitive adapter protein for high molecular weight complexes involved in regulation of catecholamine homeostasis

**DOI:** 10.1093/hmg/ddx294

**Published:** 2017-07-25

**Authors:** Dominik Piston, Lydia Alvarez-Erviti, Vikas Bansal, Daniela Gargano, Zhi Yao, Gyorgy Szabadkai, Mark Odell, M Rhyan Puno, Benny Björkblom, Jodi Maple-Grødem, Peter Breuer, Oliver Kaut, Jan Petter Larsen, Stefan Bonn, Simon Geir Møller, Ullrich Wüllner, Anthony H V Schapira, Matthew E Gegg

**Affiliations:** 1 Department of Clinical Neuroscience, UCL Institute of Neurology, London, UK; 2 Norwegian Centre for Movement Disorders, Stavanger University Hospital, Stavanger, Norway; 3 German Centre for Neurodegenerative Diseases (DZNE), Bonn, Germany; 4 Molecular Neurobiology, Centre for Biomedical Research of Rioja, Logrono, Spain; 5 Computational Systems Biology, Site Göttingen German Center for Neurodegenerative Diseases (DZNE), Göttingen, Germany,; 6 Centre for Organelle Research, University of Stavanger, Stavanger, Norway; 7 Department of Cell and Developmental Biology, Consortium for Mitochondrial Research, University College London, London, UK; 8 Department of Molecular and Applied Biosciences, University of Westminster, London, UK; 9 Department of Chemistry, Umeå University, SE-90187 Umeå, Sweden; 10 Department of Neurology, University of Bonn Medical Centre, Bonn, Germany; 11 Department of Biological Sciences, St. John's University, New York, NY, USA

## Abstract

DJ-1 is an oxidation sensitive protein encoded by the PARK7 gene. Mutations in PARK7 are a rare cause of familial recessive Parkinson’s disease (PD), but growing evidence suggests involvement of DJ-1 in idiopathic PD. The key clinical features of PD, rigidity and bradykinesia, result from neurotransmitter imbalance, particularly the catecholamines dopamine (DA) and noradrenaline. We report in human brain and human SH-SY5Y neuroblastoma cell lines that DJ-1 predominantly forms high molecular weight (HMW) complexes that included RNA metabolism proteins hnRNPA1 and PABP1 and the glycolysis enzyme GAPDH. In cell culture models the oxidation status of DJ-1 determined the specific complex composition. RNA sequencing indicated that oxidative changes to DJ-1 were concomitant with changes in mRNA transcripts mainly involved in catecholamine metabolism. Importantly, loss of DJ-1 function upon knock down (KD) or expression of the PD associated form L166P resulted in the absence of HMW DJ-1 complexes. In the KD model, the absence of DJ-1 complexes was accompanied by impairment in catecholamine homeostasis, with significant increases in intracellular DA and noraderenaline levels. These changes in catecholamines could be rescued by re-expression of DJ-1. This catecholamine imbalance may contribute to the particular vulnerability of dopaminergic and noradrenergic neurons to neurodegeneration in PARK7-related PD. Notably, oxidised DJ-1 was significantly decreased in idiopathic PD brain, suggesting altered complex function may also play a role in the more common sporadic form of the disease.

## Introduction

Parkinson’s disease (PD) is a neurodegenerative disorder characterized by typical motor symptoms including bradykinesia, rigidity and resting tremor in later stages of the disease when up to 80% of dopaminergic neurons in the brain are lost (1,2). In addition to the dopaminergic system other neurotransmitters are affected, in particular the noradrenergic system (3–5).

The PARK7 gene encodes the protein DJ-1 and mutations in this gene are a rare cause of autosomal-recessive early-onset parkinsonism. These mutations generally result in a loss-of-function of the protein (exon 1-5 deletion, L166P, L172Q). Increasing evidence suggests that there are different subcellular pools of DJ-1, with the largest proportion of DJ-1 being localised in the cytosol and minor amounts resident in mitochondria and nuclei of cultured cells and brain (6–11).

DJ-1 has been suggested to be involved in many cellular processes, including transcriptional and translational regulation, protein quality control and mitochondrial function (6,7,10,12–18). DJ-1 has also been linked to neurotransmitter homeostasis. It has been reported to affect dopamine (DA) re-uptake in HEK-293 T cells (19) and synthesis in SH-SY5Y cells (18). Dopaminergic neurons have been reported to be protected against DA toxicity by DJ-1 via control of the vesicular sequestration of DA and upregulation of VMAT2 (20). Conversely, DJ-1 deficiency impairs the expression of neurotransmitter receptors and neurotransmission (21,22).

There is common agreement that DJ-1 acts as a sensor for oxidative stress and that its reactive cysteine residues C46, C53, and C106 are involved in the protein’s regulation. It has been suggested that C106 is the most sensitive residue to oxidation and might therefore act as a molecular switch for the activity of the protein (7,23). Since DJ-1 has clearly been linked to oxidative stress, which represents one of the key features of PD on the molecular level (24), understanding the protein’s role in cellular stress response might provide new insights into the processes underlying sporadic forms of the disease. Moreover, understanding the effects of loss-of-function of DJ-1 might identify new therapeutic strategies and reveal novel mechanisms critical for disease pathogenesis and/or early events resulting in neuronal death.

Several studies have shown that wild type DJ-1 is part of high molecular weight (HMW) complexes in human brain as well as in cultured cells. Different sizes have been observed for the described complexes, ranging from 70 kDa up to 2 MDa with conflicting protein constituents (25–28). Some of the specific functions of DJ-1-containing HMW protein complexes include protein degradation via the ubiquitin-proteasome-system (28), prevention of alpha-synuclein accumulation (15) and RNA regulation (29,30). We hypothesised that DJ-1 binds various proteins to form HMW complexes that can regulate the function/localisation of these proteins within cells and furthermore that the oxidation status of DJ-1 might orchestrate the composition of these complexes.

Here, we show in human neuroblastoma cells and brain that DJ-1 forms HMW complexes, and that DJ-1 loss of function resulted in transcriptional dysregulation of genes involved in neurotransmitter synthesis, transport, storage and release. Loss of DJ-1 complexes *in vitro* increased intracellular catecholamine levels in human neuroblastoma cells and may provide an insight for the role of DJ-1 in PD pathogenesis.

## Results

### Levels of dimeric and oxidized monomeric DJ-1 are decreased in PD brain

**Figure 1.**
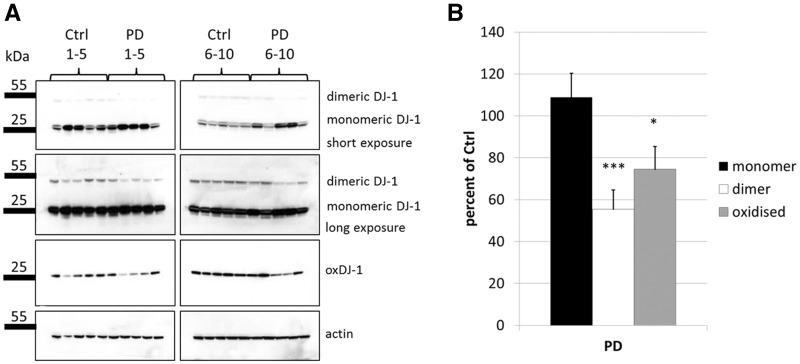
Levels of dimeric and oxidised DJ-1 are altered in human PD brain. (**A**) SDS PAGE and western blot analysis of human post mortem tissue lysates from 10 control individuals versus 10 PD patients. Membranes were probed for total DJ-1 (Enzo mouse monoclonal) to detect monomeric and dimeric forms of the protein. An antibody that detects oxidized DJ-1 at residue C106 (oxDJ-1) was also used. β-actin was used as a loading control. (**B**) Quantification of the western blots shown in (A). Statistical significance was calculated by one-way ANOVA with post hoc Tukey HSD test. *P < 0.05; ***P < 0.01 vs. control.

Several groups have reported altered levels of total or oxidised forms of DJ-1 in human post mortem tissue of PD patients, although results are not consistent (31–33). We performed SDS PAGE and western blotting analysis on post mortem tissue from cortex of age-matched controls (*n = *10) and PD patients (*n = *10) using two different antibodies to differentiate between monomeric, dimeric and DJ-1 oxidised at cysteine residue 106 (C106; (34)) (Fig. 1A). Levels of monomeric DJ-1 varied between individuals, but no significant difference was observed between controls and PD patients. However, levels of oxidised DJ-1 (oxDJ-1) were significantly decreased in PD brains by 25.5% (*P < *0.01) (Fig. 1B). Similarly, SDS-resistant dimeric DJ-1 was decreased by 44.5% in PD brains (*P < *0.05). The oxDJ-1 antibody did not detect dimeric DJ-1, suggesting that DJ-1 dimers are not oxidised. However, it cannot be discounted that the epitope is concealed in the dimeric form or that the levels of dimeric compared to monomeric were below the detection limit of the antibody. In summary, alterations in the levels of oxidised and dimeric DJ-1 appeared to be associated with PD.

### Complex bound DJ-1 is the predominant form of the protein in human brain

**Figure 2.**
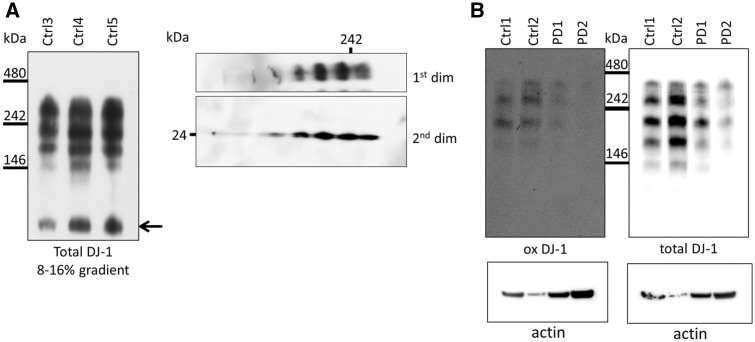
DJ-1 HMW complexes are the predominant species in human brain and show evidence of oxidation. (**A**) Left: Soluble complexes from putamen of three healthy individuals were examined using 8-16% gradient BisTris gels and subsequent western blot analysis for total DJ-1. No monomeric DJ-1 was detectable in putamen in native lysates. However, based on its molecular weight a band perhaps representing DJ-1 homodimer was detected (indicated by arrow). Representative western blot is shown (*n = *3). Right: The soluble complexes from human putamen were analysed by two dimensional BN/SDS PAGE and western blotting for total DJ-1, which was detectable in all four complexes, illustrating that the DJ-1 signal is specific. Representative western blot is shown (*n = *2). (**B**) Soluble complexes from cortex of two healthy individuals and two PD patients were separated on 7.5% fixed percentage BisTris gels and subsequently probed for oxidized DJ-1 (ox DJ-1) and total DJ-1. Representative western blot is shown (*n = *2). Protein loading of brain samples used for BN-PAGE was assessed by measuring an aliquot of each sample for β-actin by SDS-PAGE (panel underneath BN PAGE).

We hypothesised that participation of DJ-1 in the formation of variable complexes could account for its numerous reported functions. To detect soluble DJ-1 containing HMW complexes in human post mortem tissue (putamen) we performed blue native (BN) PAGE. Western blotting identified several DJ-1 complexes (Fig. 2A, left panel) including dimer (Fig. 2A, arrow). To confirm that these HMW species contained DJ-1 we performed combined two-dimensional BN and SDS PAGE. The HMW complexes all yielded monomeric DJ-1 at the expected molecular weight (Fig. 2A, right panel). A similar pattern of complexes was detected in the cortex (Fig. 2B), amygdala and substantia nigra (Supplementary Material, Fig. S1). The total amount of DJ-1 complexes in the putamen was significantly increased in PD brains (Supplementary Material, Fig. S1A; *P < *0.01), while there was no difference in the amygdala or substantia nigra. It should be noted that the same pattern of complexes was seen independently of post mortem delay (range: 3–89.2 h) suggesting that this pattern was not due to degradation following extraction. Furthermore, these complexes appear very stable as complexes extracted from cells or human brain could be kept in sucrose buffer at 4 °C for up to two weeks and still yield the same western blot pattern (data not shown).

Due to the different cell-types in brain, the observed pattern of DJ-1 complexes in human brain could reflect cell-type specific complexes. Alternatively, the different complexes might reflect the oxidation state of DJ-1 at C106. Therefore, we probed the DJ-1 complexes in control and PD cortex with oxDJ-1 antibody and observed that all HMW species were positive for oxidised DJ-1 (Fig. 2B). The two PD cortex samples suggest that there are less DJ-1 positive HMW complexes than in control brains.

### Complex bound DJ-1 is the predominant species also in SH-SY5Y cells

**Figure 3.**
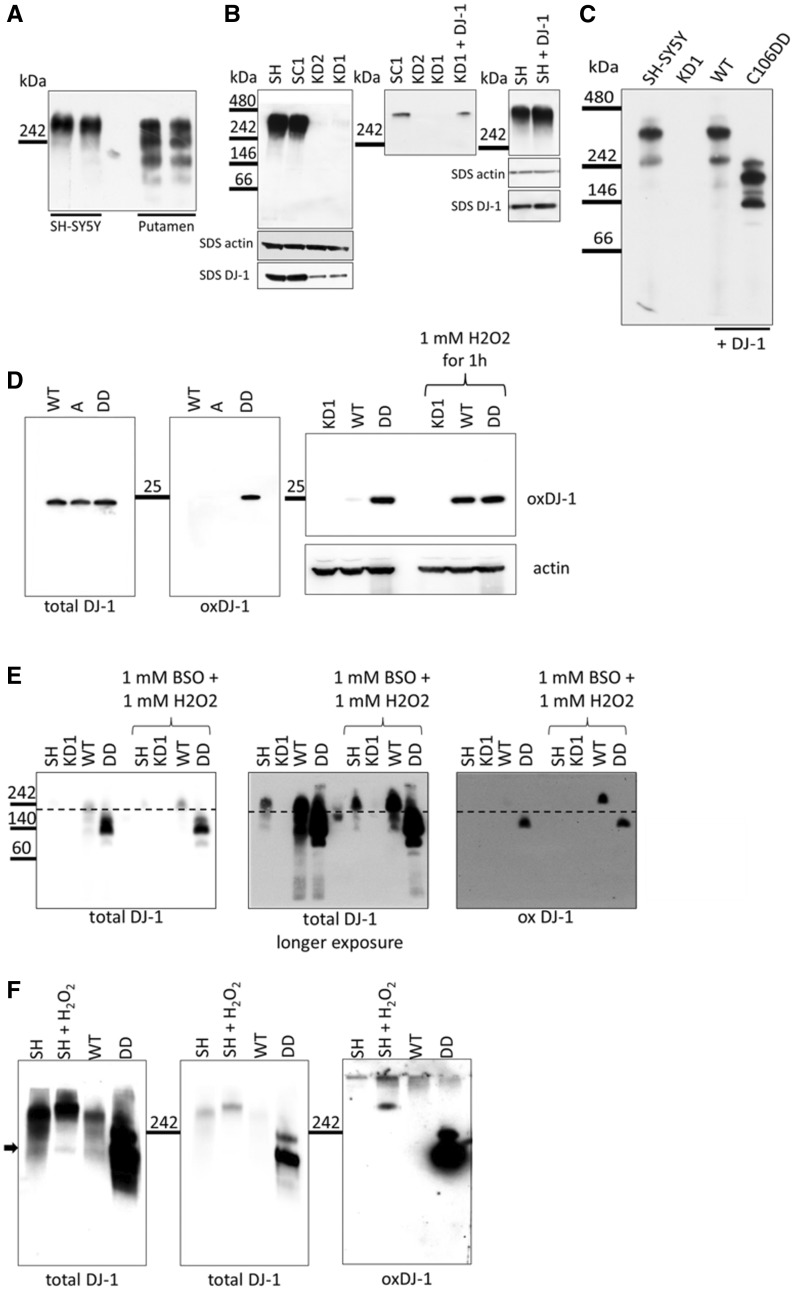
Oxidation of DJ-1 affects complex formation. (**A**) Soluble complexes from SH-SY5Y cells and control human putamen brain samples were separated by BN PAGE using 7.5% BisTris gels and western blot analysis for DJ-1 was performed. (**B**) Left: Soluble protein complexes were extracted from SH-SY5Y, scrambled control (SC1), DJ-1 knock down (KD1 and KD2) cells and separated by BN PAGE on 7.5% BisTris gels. Middle: Transfection of WT DJ-1 into the KD background rescued complex formation after 72 h. Right: Over expression of WT DJ-1 in SH-SY5Y cells slightly increased the amount of complex after 72 h compared to untransfected SH-SY5Y cells. Representative western blots are shown (*n > *3). (**C**) To compare total amounts of monomeric, dimeric and complex bound DJ-1, soluble protein complexes were extracted from SH-SY5Y cells, KD1 and KD1 cells transfected with WT DJ-1 or C106DD DJ-1 72 h after transfection. Complexes were separated on 8-16% gradient BisTris gels and western blot analysis was performed probing for DJ-1. Representative western blot results are shown (*n > *3). Protein loading of samples used for BN-PAGE was assessed by measuring an aliquot of each sample for DJ-1 and β-actin level by SDS-PAGE (panels underneath BN PAGE). (**D)** Left: SDS PAGE and western blot analysis was performed with KD1 cells transiently transfected with wild type DJ-1 (WT), the oxidation deficient mutant DJ-1 form C106A (A) or the oxidation mimicking DJ-1 form C106DD (DD). Membranes were probed for oxidised DJ-1 (oxDJ-1) and total DJ-1. Right: SDS PAGE and western blot analysis was performed with KD1 cells, untransfected or transiently transfected with WT or DD DJ-1 Cells were either untreated or stress treated with 1 mM H_2_O_2_ for 1 h to induce oxidation of DJ-1. Membranes were probed for oxidised DJ-1 (oxDJ-1) and β-actin. (**E)** Soluble protein complexes were extracted from SH-SY5Y cells, KD1, KD1 expressing WT or C106DD DJ-1and separated by BN PAGE on 8-16% BisTris gels. Cells were either stress treated with 1 mM BSO for 24 h and subsequently 1 mM H_2_O_2_ for 30 min or left untreated. Left: short exposure probing for total DJ-1. **Middle:** longer exposure of the same membrane. **Right:** Membrane probed for oxidised DJ-1 (ox DJ-1). The dotted line denotes the molecular weight of WT DJ-1 complex under basal conditions and indicates that treatment induced oxidation of DJ-1 results in a slightly higher molecular weight of the complex (**F)** Soluble protein complexes were extracted from SH-SY5Y cells either stress treated (1 mM BSO for 24 h and subsequently 1 mM H_2_O_2_ for 30 min) or left untreated. Samples were separated by BN PAGE on 8-16% BisTris gels and western blot analysis for total and oxidised DJ-1 was performed. Left: longer exposure probing for total DJ-1. Middle: shorter exposure of the same membrane. Right: Membrane probed for oxidised DJ-1. The arrow indicates another complex detected by total DJ-1 antibody in oxidation treated SH-SY5Y cells with the same molecular weight as C106DD DJ-1 complex.

To investigate if DJ-1 forms similar HMW complexes in cultured cells, we analysed DJ-1 complex formation in the human neuroblastoma SH-SY5Y cell line by BN PAGE. Western blotting using antibody that labels total DJ-1 identified one DJ-1 complex with a similar molecular weight to the largest complex in the human brain (Fig. 3A). The HMW complex was almost absent in the constitutive DJ-1 knock down (KD) cell lines KD1 and KD2 (Fig. 3B left panel), when compared to the parental SH-SY5Y cell line (SH) or SH-SY5Y expressing scrambled control shRNA (SC1). Analysis of total DJ-1 protein levels by SDS-PAGE and western blotting indicated that < 20% endogenous DJ-1 is expressed in KD cells compared to the SH or SC1 cell lines (Supplementary Material, Fig. S2A and B). Re-introduction of wild type (WT) DJ-1 rescued complex formation, while over expression of WT DJ-1 in normal SH-SY5Y cells resulted in increased complex levels (Fig. 3B middle and right panel).

DJ-1 is predominantly localised in the cytosol but there is also a mitochondrial DJ-1 pool (7,8,13). Subcellular fractionation of SH-SYSY cells followed by BN-PAGE showed that the HMW DJ-1 complex was present in the cytosolic fraction (Supplementary Material, Fig. 2C). Although BN-PAGE did not detect DJ-1 HMW complex in mitochondria, SDS-PAGE followed by Western blotting indicated that wild-type DJ-1 can be detected in both cytosolic and mitochondrial fractions, as previously described (7,8).

In human brain material, complex bound DJ-1 was the predominant form of the protein (Fig. 2). Therefore, we investigated the proportions of monomeric, dimeric and complex bound DJ-1 in SH-SY5Y cells by BN PAGE using gradient gels. The increased resolution indicated that there are at least two HMW complexes containing DJ-1 in SH-SY5Y cells and WT cells within a size range of 242 and 480 kDa (Fig. 3C). A minor signal at around 50 kDa which could represent the DJ-1 homodimer was only detectable upon extended exposure of the western blots (Supplementary Material, Fig. S3A).

### Oxidation state of DJ-1 affects complex formation

We next investigated the impact of C106 oxidation on complex formation in SH-SY5Y cells. Mutation of cysteine 106 to alanine (C106A) prevents oxidation of this residue, while mutation of the same residue to an aspartic acid doublet (C106DD) is thought to mimic sulfinic acid oxidative modification (-SO_2_H) and has been shown to be critical for DJ-1 function (12). WT or C106DD plasmids were transiently transfected into the DJ-1 KD cell line KD1 to create cells predominantly expressing the DJ-1 form of interest. Note that the siRNA in KD1 targets the 3’UTR of endogenous DJ-1 mRNA and thus plasmid encoded WT or C106DD DJ-1 mRNA transcripts were unaffected. BN PAGE revealed that transfection of KD1 cells with C106DD resulted in the formation of at least three different complexes of lower molecular weight that were closer to 242 kDa (Fig. 3C). The oxidation-mimic C106DD was readily detected by the oxDJ-1 antibody by SDS-PAGE, while WT-DJ-1 was detected only after oxidative stress treatment (1 mM BSO for 24 h and 1 mM H_2_O_2_ for 1 h; Fig. 3D), indicating that DJ-1 C106DD protein adequately mimics endogenous DJ-1 oxidized at C106.

Similar results were observed in KD1 cells with stable expression of WT or C106DD DJ-1 cells. WT and DD stable cell lines expressed 4.1 and 5.6-fold more DJ-1 than parental SH-SY5Y cells, respectively (Supplementary Material, Fig. S3D). The oxDJ-1 antibody also detected C106DD in HMW DJ-1 complexes separated by BN-PAGE (Fig. 3E), whereas WT DJ-1 was detected only following oxidative stress treatment (Fig. 3E, right panel). The dotted line in Figure 3E denotes the molecular weight of complexes containing WT DJ-1 under basal conditions and indicates that treatment induced oxidation of DJ-1 results in a higher molecular weight of the complex. The lower molecular weight forms of these complexes seen in C106DD cells were not detected following induction of oxidative stress in cells expressing WT DJ-1. Probing for total DJ-1 confirmed that WT DJ-1 complexes slightly shifted up following oxidative stress (Fig. 3E, middle panel). Treatment of parental SH-SY5Y cells with the same oxidising conditions also resulted in a modest increase in HMW DJ-1 complex size (Fig. 3F). The arrow in Figure 3F indicates another complex in treated SH-SY5Y cells with the same molecular weight as observed upon expression of DJ-1 C106DD.

These results demonstrate that oxidation of DJ-1 affects the size and probably the composition of DJ-1 HMW complexes. However, the HMW complexes formed by the oxidation-mimic C106DD DJ-1 migrate differently compared to oxidised endogenous DJ-1 HMW complexes. It cannot be excluded that the extra aspartic acid residue may affect the charge of the protein and hence it’s migration through the gel.

### DJ-1 complexes are absent upon expression of the PD associated DJ-1 mutant L166P

**Figure 4.**
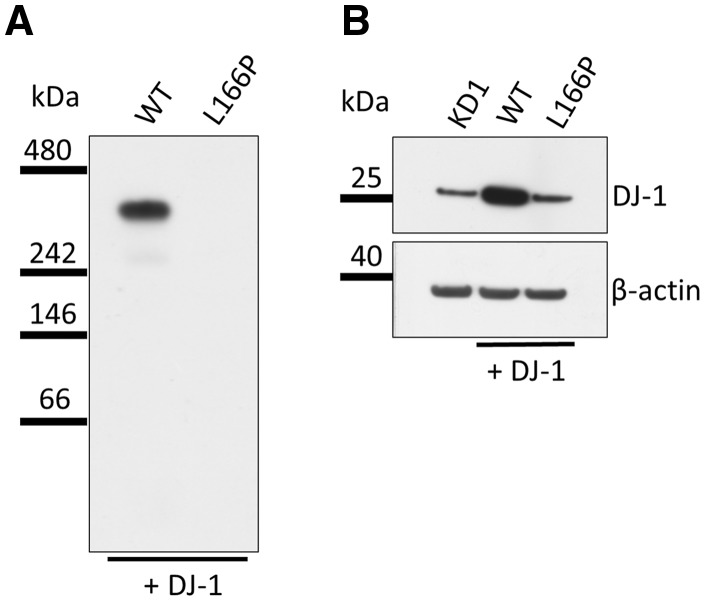
DJ-1 complexes are not detected upon expression of the PD associated DJ-1 mutant L166P. (**A**) Soluble protein complexes were extracted from KD1 cells transfected with WT DJ-1 or L166P DJ-1 72 h after transfection. Complexes were separated on 8–16% gradient BisTris gels and western blot analysis was performed probing for DJ-1. In cells expressing the PD associated mutation DJ-1 L166P the complex was absent. Representative western blot results are shown (*n = *2). (**B**) Analysis of total DJ-1 levels in the same samples by SDS PAGE and western blotting revealed that introduction of WT-DJ-1 in to KD1 cells significantly increased total DJ-1 levels. However total DJ-1 levels in KD1 cells expressing DJ-1 L166P were almost as low as in KD1 cells alone. β-actin was used as loading control. Representative western blot is shown.

The PD-associated mutation DJ-1 L166P has been shown to be less stable resulting in its premature proteasomal degradation (35,36). In KD1 cells transiently transfected with DJ-1 L166P HMW protein complexes were absent (Fig. 4A). To ensure that the L166P DJ-1 protein was expressed following transfection, we performed SDS PAGE and western blotting. Protein levels were substantially reduced (Fig. 4B). To ensure efficient transfection, mRNA levels were analysed by quantitative real-time PCR (qPCR). qPCR results confirmed substantially increased DJ-1 mRNA levels (SH-SY5Y, 100 ± 5%; KD1, 9 ± 23%; KD1 + WT DJ-1, 173864 ± 12%; KD1 + L166P DJ-1, 65682 ± 15%). Therefore, in our experimental model, DJ-1 L166P is unstable and results in protein levels similar to KD1 cells and thus were unable to detect HMW DJ-1 complexes in these cells.

### Composition of DJ-1 HMW complexes

Our experiments suggested altered complex composition of DJ-1 complexes in an oxidation dependent manner (Fig. 3). Immunoprecipitation of DJ-1 WT HMW complexes from SH-SY5Y cells was performed to analyse the composition of the complexes by label-free quantitative liquid chromatography tandem mass spectrometry (LC-MS/MS; (37)). A band of the correct molecular weight was detected following immunoprecipitation with DJ-1 antibody and was barely detectable or absent when the immunoprecipitation was performed in either KD1 cells or without DJ-1 antibody, respectively (Supplementary Material, Fig. S4A). Identified proteins are listed in Supplementary Material, Table S1. DJ-1 was present in all four runs and the second most common protein. The total number of DJ-1 peptides was 62 with 94% coverage of the protein. The identified proteins included Glyceraldehyde 3-phosphate dehydrogenase (GAPDH), Hsp60, SOD1 and three isoforms of heterogeneous ribonucleoproteins (A1, C1C2 and A2B1), the putative elongation factor 1 alpha like 3 (EF1A3) and the splicing regulatory glutamine lysine rich protein 1 (SREK1). Note that trypsin is from the mass spectrometry protocol and yeast enolase is used as a control.

BN-PAGE of SH-SY5Y lysates followed by western blotting for hnRNPA1 indicated that it was present in a HMW complex with a similar migration to DJ-1 (Supplementary Material, Fig. S5A). Furthermore immunoprecipitation of hnRNPA1 from SH-SY5Y cells co-immunoprecipitated DJ-1 (Supplementary Material, Fig. S5B). Two-dimensional BN and SDS-PAGE also indicated that hnRNPA1 and GAPDH co-migrated with DJ-1 (Supplementary Material, Fig. S5C and F).

To elucidate the effect of oxidative stress we compared DJ-1 HMW complexes obtained from KD1 cells expressing DJ-1 C106A and DJ-1 C106DD by the same LC-MS/MS technique. We chose C106A rather than the parental cell line or KD1 cells expressing WT-DJ-1, since C106A is unable to be oxidised at this residue. We did not analyse endogenous complexes treated with oxidising conditions (Fig. 3C and F) as it is likely that several other residues, in addition to C106, are oxidised under these conditions. Equal expression levels of C106A and C106DD DJ-1 in the cells used for this experiment were confirmed by SDS PAGE and western blot (Supplementary Material, Fig. S4B). The differences identified between the two samples are listed in Supplementary Material, Table S2 together with their mean amount in fmol and the respective fold change between the samples for each protein (C106DD/C106A). The greatest change was a five-fold reduction of heterogeneous ribonucleoprotein A1 like 2 (hnRNPA1L2; 19.9%) in the complex containing DJ-1 C106DD. hnRNPA1L2 has 97% protein homology to hnRNPA1, which was also found to be decreased (58%). Several additional proteins associated with RNA metabolism, such as other hnRNPs family members (A2B1, A3, A0, C1C2Q, D0, F, H, K, L, M, R, U) and polyadenylate-binding protein 1 (PABP1; decreased by 54%) were found to be altered in C106DD cells. GAPDH (also found in initial mass spectrometry of endogenous DJ-1 HMW complexes) was also slightly decreased, as was hsc70 (also known as hsp7c), a protein involved in chaperone-mediated autophagy of α-synuclein, and reported to be decreased in sporadic PD (38).

BN-PAGE analysis for PABP1 indicated that this protein was present in WT DJ-1 HMW complexes. Furthermore, a PABP1 signal was detected in cells expressing C106DD DJ-1 at the expected lower molecular weight (Supplementary Material, Fig. S5D). Two-dimensional BN and SDS-PAGE indicated that both hnRNPA1 and PABP1 co-migrated with DJ-1 in KD1 cells expressing WT-DJ-1 (Supplementary Material, Fig. S5C and E). The hnRNP1 spot was decreased in cells expressing C106DD (Supplementary Material, Fig. S5C). Two PABP1 spots of the correct molecular weight were detected that co-migrated with DJ-1 (Supplementary Material, Fig. S5E). In cells expressing C106DD DJ-1 one of these spots was not detected. GAPDH and hsc70 also co-migrated with DJ-1 (Supplementary Material, Fig. S5F). In summary, DJ-1 complexes contained largely RNA binding proteins and the composition appears to be altered by the oxidation status of DJ-1.

### Transcriptome alterations are consistent with altered DJ-1 complex composition

We next wanted to understand the effect of oxidation of DJ-1 on complex bound RNA binding proteins. Analysis of small RNAs (miRNA; piRNA; rRNA; snoRNA); small nuclear RNA (snRNA)) and larger RNAs (mainly mRNA) by RNA sequencing revealed differences in miRNA and mRNA species only. All other small RNA species were largely unaffected. The mapping statistic for the RNA sequencing is shown in Supplementary Material, Table S3. We correlated changes in miRNAs with the respective changes in mRNA in order to extract inversely correlated miRNA-mRNA pairs, to increase the overall reliability of the data. The top hits of inversely correlated miRNA-mRNA pairs are shown in Table 1. These included several neurotransmitter associated targets such as Vesicular monoamine transporter 1 (SLC18A1/VMAT1), Solute Carrier Family 1 Neuronal/Epithelial High Affinity Glutamate Transporter (SLC1A1), and Dopamine receptor 2 (DRD2). Notably one of the top hits was apoptosis signaling kinase 1 (MAP3K5/ASK1), which has previously been shown to be partially activated in cells expressing C106DD DJ-1 and to be cytoprotective (12). Assessment of gene ontology (GO) terms for both up regulated and down regulated mRNA species is shown in Supplementary Material, Figure S8. Biological regulation, metabolic process, membrane, nucleus, vesicle, and protein binding featured strongly for both up and down regulated mRNA.

**Table 1.** Top up and down regulated mRNA targets in DD cells when compared to WT cells that showed inverse correlation with their respective miRNAs

Gene	Description	Pathway	log2 fold change	*P*-value
Down regulated				
SGK1	Serum and glucocorticoid-regulated kinase 1	HD signaling	−2.73	1.47E-68
TGM2	Tissue transglutaminase 2	HD signaling	−2.28	2.66E-75
ITGA5	Integrin alpha-5	Apoptosis	−2.16	3.43E-09
CPLX2	Complexin-2	HD signaling	−1.94	6.17E-36
SYK	Spleen tyrosine kinase	Inflammation	−1.87	8.50E-36
PRKCA	Protein kinase C alpha type	Apoptosis	−1.76	5.36E-157
GRIP1	Glutamate receptor-interacting protein 1	Glutamate receptor signaling	−1.71	3.12E-48
GRIN1	Glutamate receptor ionotropic, NMDA 1	Dopamine/cAMP signaling; Glutamate receptor signaling	−1.56	4.00E-03
CAPN6	Calpain-6	Apoptosis	−1.54	4.05E-05
KCNJ3	G protein-activated inward rectifier potassium channel 1	Dopamine/cAMP signaling	−1.44	1.83E-02
GRM2	Metabotropic glutamate receptor 2	Glutamate receptor signaling	−1.31	3.19E-02
CACNA1C	Voltage-dependent L-type calcium channel subunit alpha-1C	Dopamine/cAMP signaling	−1.01	4.08E-04
GRM7	Metabotropic glutamate receptor 7	Glutamate receptor signaling	−0.89	1.18E-11
Up regulated				
SLC18A1/ VMAT1	Solute carrier 18A1/vesicular monoamine transporter 1	Dopamine receptor signalling	1.66	1.75E-229
MAP3K5/ ASK1	Mitogen-activated protein kinase kinase kinase 5/Apoptosis signal-regulating kinase 1	Apoptosis	1.16	4.64E-20
CYGB	Cytoglobin	Superoxide radical degradation	1.04	1.29E-29
MYCN	N-myc proto-oncogene protein	ERK/MAPK signaling	0.94	7.32E-10
HSPA1A	Heat shock 70 kDa protein 1A	HD signaling	0.76	7.59E-08
CCND1	Cyclin-D1	cell cycle control (PI3/Akt)	0.63	5.77E-42
MAPK11	Mitogen-activated protein kinase 11	PD signaling	0.63	9.45E-11
SLC1A1	Solute carrier 1A1/Excitatory amino acid transporter 3	Glutamate receptor signaling	0.61	1.78E-05
THEM4	Acyl-coenzyme A thioesterase	Cell cycle control (PI3/Akt)	0.55	1.92E-06
BMPR1B	Bone morphogenetic protein receptor type-1B	PTEN signaling	0.48	3.92E-09
Hsc70	Heat shock 70 kDa protein 8/Heat shock cognate 71 kDa protein	HD signaling	0.46	3.27E-16
DRD2	Dopamine receptor D2	Dopamine receptor signalling; Dopamine/cAMP signaling	0.45	7.31E-04
KCNJ8	ATP-sensitive inward rectifier potassium channel 8	Dopamine/cAMP signaling	0.44	7.68E-07
PIK3R1	Phosphatidylinositol 3-kinase regulatory subunit alpha	HD signaling	0.43	1.26E-07
RPS24	40S ribosomal protein S24	PD signaling	0.38	6.27E-03
IFT57	Intraflagellar transport protein 57 homolog	HD signaling	0.38	6.99E-07

**Figure 5.**
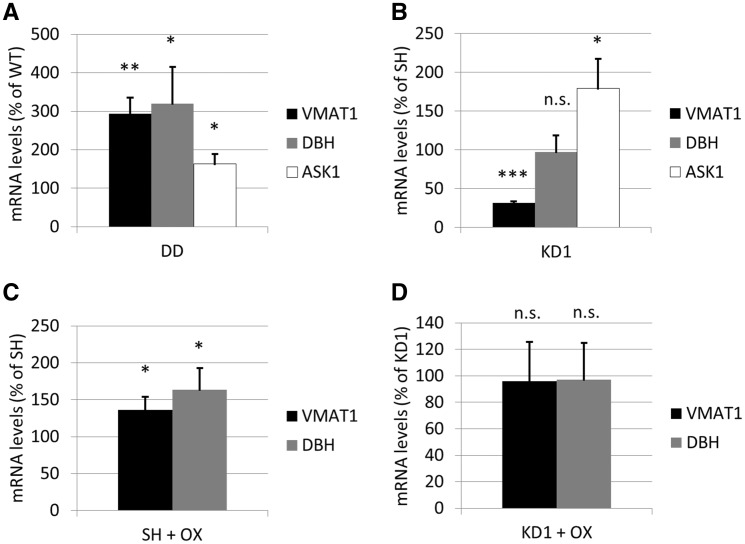
VMAT1, DBH and ASK1 mRNA in oxidized and DJ-1 KD cells. qPCR was performed on SH-SY5Y cells, KD1, WT and DD cells and relative expression calculated. Data were normalized to β-actin mRNA levels. (**A**) mRNA levels in DD cells are expressed as % WT levels (*n = *4–5). (**B**) KD1 mRNA levels are expressed as % SH-SY5Y cells (*n = *4–5). (**C**) SH-SY5Y cells treated with oxidative stress (1 mM BSO for 24 h and 1 mM H_2_O_2_ for 30 min; OX) and expressed as % of untreated SH-SY5Y (*n = *3–5). (**D**) mRNA levels in KD1 cells treated with oxidative stress and expressed as % untreated KD1 cells (*n = *3–5). Statistical significance calculated using Students T-test versus respective control. *P < 0.05 vs. SH/WT; **P < 0.01 vs. WT; ***P < 0.001 vs. SH; n.s., non-significant.

Differentially regulated mRNAs (without correlation to miRNAs) were related to synthesis, transport, release & metabolism of neurotransmitters, especially catecholamines and glutamate. These included dopa decarboxylase (DDC), VMAT1 and dopamine β hydroxylase (DBH) (Table 2). Confirming the RNA sequencing data, qPCR of VMAT1, DBH and ASK1 mRNA showed significantly increased levels in cells expressing DJ-1 C106DD when compared to expression of DJ-1 WT (Fig. 5A; VMAT1: 3-fold, *P < *0.01; DBH: 3.2-fold, *P < *0.05; ASK1: 1.6-fold, *P < *0.05). In DJ-1 KD cells (KD1), VMAT1 mRNA levels were significantly decreased by 3.2-fold (*P < *0.001), whereas DBH mRNA levels remained unchanged and ASK1 mRNA levels increased 1.8-fold, *P < *0.05 (Fig. 5B). Oxidative stress treatment of parental SH-SY5Y cells significantly increased both VMAT1 (1.3-fold, *P < *0.05) and DBH mRNA levels (1.6-fold, *P < *0.05; Fig. 5C), whereas KD1 cells lacking DJ-1 were unable to increase either transcript (Fig. 5D). In summary, RNA sequencing and qPCR suggest that alterations of DJ-1 HMW complex composition are concomitant with changes in the transcription of several neurotransmitter-related targets.

**Table 2.** Top up and down regulated mRNA targets in DD cells when compared to WT cells irrespective of miRNAs

Gene	Description	log2 fold change	*P*-value
Down regulated				
** MOXD1**	Monooxygenase, DBH-like 1	−4.47	4.37E-55
** CORIN**	Corin, serine peptidase	−4.24	5.53E-30
** GABRB3**	Gamma-aminobutyric acid (GABA) A receptor, beta 3	−4.17	1.13E-41
** PPP2R2C**	Protein phosphatase 2, regulatory subunit B, gamma	−3.88	1.24E-12
** HTR2B**	5-Hydroxytryptamine (serotonin) receptor 2B, G protein-coupled	−2.94	3.41E-14
** DKK2**	Dickkopf WNT signaling pathway inhibitor 2	−2.77	1.23E-294
** SGK1**	Serum/glucocorticoid regulated kinase 1	−2.73	1.47E-68
** SLC18A3**	Solute carrier family 18 (vesicular acetylcholine) 3	−2.54	6.83E-12
** GAD1**	Glutamate decarboxylase 1 (brain, 67kDa)	−2.44	4.81E-34
** GRM8**	Glutamate receptor, metabotropic 8	−2.35	3.52E-05
** CHRM2**	Cholinergic receptor, muscarinic 2	−2.3	1.15E-11
** PLK2**	Polo-like kinase 2	−2.18	4.14E-79
** MAOA**	Monoamine oxidase A	−0.43	2.98E-08
Up regulated				
** DDC**	Dopa decarboxylase (aromatic L-amino acid decarboxylase)	1.94	3.16E-287
** IGF2**	Insulin-like growth factor 2 (somatomedin A)	1.93	1.04E-71
** MIR143HG**	MIR143 host gene (non-protein coding)	1.89	1.95E-03
** DBH-AS1**	DBH antisense RNA 1	1.77	1.01E-53
** SLC18A1**	Solute carrier family 18 (vesicular monoamine) 1	1.66	1.75E-229
** HTR1E**	5-Hydroxytryptamine (serotonin) receptor 1E, G protein-coupled	1.54	5.07E-03
** DBH**	Dopamine beta-hydroxylase (dopamine beta-monooxygenase)	1.35	6.73E-201
** SLC6A2**	Solute carrier family 6 (neurotransmitter transporter, noradrenalin) 2	0.64	6.07E-49
** SLC1A1**	Solute carrier family 1 (neuronal/epithelial high affinity glutamate transporter) 1	0.61	1.78E-05
** DRD2**	Dopamine receptor D2	0.45	7.31E-04

### KD of DJ-1 increased catecholamine levels

**Figure 6.**
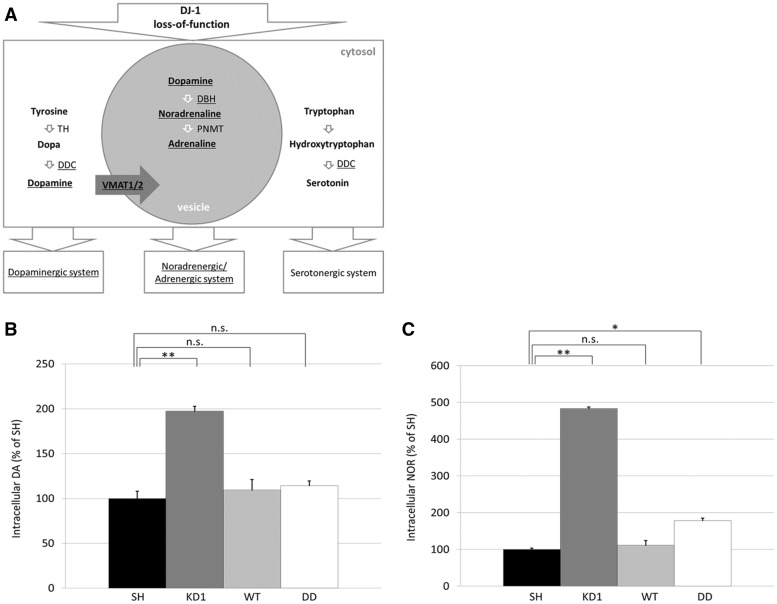
Knock down of DJ-1 affects the dopaminergic and noradrenergic system. **(A**) Overview of catecholamine synthesis pathways. Abbreviations: tyrosine hydroxylase (TH), dopa decarboxylase (DDC), dopamine beta hydroxylase (DBH), phenylethanolamine-N-methyltransferase (PNMT). (**B,C**) Results of catecholamine ELISA to measure intracellular levels of dopamine (DA) and noradrenaline (NOR). Catecholamine levels were calculated as pg/mg protein. Results are expressed as percent of SH-SY5Y cells (*n = *3). Statistical significance was determined by one-way ANOVA with post hoc Tukey HSD test. *P < 0.05 vs. SH; **P < 0.01 vs. SH; n.s., non-significant.

Several of the upregulated transcripts are directly involved in the synthesis of DA (DDC), transport of DA into vesicles (VMAT1) and its metabolism to noradrenaline (DBH; Fig. 6A). We thus measured the levels of intracellular DA (Fig. 6B) and noradrenaline (NOR; Fig. 6C) in our cell models by ELISA. KD of DJ-1 in KD1 cells increased DA levels 2-fold compared to normal SH-SY5Y cells (KD1, 11.83 ± 0.65 pg/mg protein; SH, 5.99 ± 0.49 pg/mg protein; *P < *0.01). The constitutive expression of WT or C106DD DJ-1 in KD1 cells rescued the KD phenotype by significantly decreasing DA levels, compared to KD1 (WT, 6.56 ± 0.77 pg/mg protein; DD, 6.85 ± 0.37 pg/mg protein). Similar results were obtained for NOR. When compared to SH-SY5Y cells, intracellular NOR levels in KD1 cells were increased 4.8-fold (KD1, 342.29 ± 14.74 pg/mg protein; SH, 70.82 ± 2.37 pg/mg protein, *P < *0.01), while re-introduction of WT or C106DD DJ-1 reduced NOR levels compared to KD1 cells (WT, 78.81 ± 9.85 pg/mg protein and DD, 126.35 ± 7.88 pg/mg protein). These results demonstrate that re-introduction of DJ-1, irrespective of whether WT or C106DD, is sufficient to rescue this phenotype and that cellular levels of DJ-1 are critical to balance catecholamine levels. Adrenaline (AD) levels were increased in KD1, and were not reversed by expression of WT or C106DD DJ-1 (Supplementary Material, Fig. S6).

**Figure 7.**
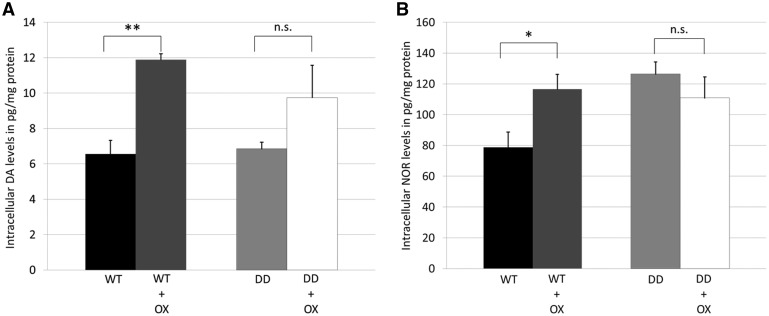
Treatment of cells with oxidative stress increased DA and NOR levels. **(A,B**) Catecholamine ELISAs to measure intracellular levels of noradrenaline (NOR) and dopamine (DA) in WT and DD cells following treatment with oxidative stress (OX; 1 mM BSO for 24 h and 1 mM H_2_O_2_ for 30 min). Catecholamine levels were calculated as pg/mg protein (*n = *3). Statistical significance determined by Students T-test compared to respective control cell line. *P < 0.05; **P < 0.01; n.s., non-significant.

We further analysed the effect of oxidative stress treatment (OX; L-BSO + H_2_O_2_) on catecholamine levels (Fig. 7). DA levels in WT cells increased 1.8-fold following treatment (WT, 6.56 ± 0.77 pg/mg protein vs. WT + OX, 11.89 ± 0.33 pg/mg, Fig. 7A;*P < *0.01), while in DD cells DA levels did not increase significantly following treatment (Fig. 7A).

NOR levels in WT cells were also significantly increased following treatment (WT, 78.81 ± 9.86 pg/mg protein; WT + OX, 116.54 ± 9.58 pg/mg, *P < *0.05). NOR levels in DD cells were similar to treated WT cells (Fig. 7B) and did not change after treatment (DD, 126.35 ± 7.88 pg/mg protein; DD + OX, 110.82 ± 13.63 pg/mg protein). Oxidative stress treatment of parental SH-SY5Y cells also increased DA levels by 3.81-fold (SH-SY5Y; 5.99 ± 0.49 pg/mg protein vs SH-SY5Y + OX; 22.82 ± 1.55 pg/mg protein), but not NOR or AD (Supplementary Material, Fig. S7).

## Discussion

PARK7-related PD is autosomal recessive caused by DJ-1 loss-of-function. We report that DJ-1 forms HMW complexes in human brain and SH-SY5Y cells. Analysis of these complexes in SH-SY5Y cells indicated that they contain RNA-related proteins such as PABP1 and hnRNPA1. KD of DJ-1 meant these complexes were not formed, and was accompanied by alterations of mRNA transcripts important for the catecholaminergic system and increased intracellular levels of DA and NOR. DJ-1 HMW complexes could not be detected in cells expressing the PD-associated DJ-1 mutation L166P.

### DJ-1 structure in sporadic and PARK7-related PD

Decreased levels of DJ-1 mRNA, monomeric and oxidized forms of DJ-1 have been observed in PD patients in several previous studies (31–33). In human PD brain we found total protein levels of monomeric DJ-1 were unaffected in all regions tested. Levels of oxidized monomeric and unoxidized dimeric DJ-1 however were significantly decreased in sporadic PD patients compared to controls. These data suggest that the decrease in oxidized monomeric and unoxidised dimeric DJ-1 might be of relevance to PD pathophysiology.

DJ-1 has been observed to dimerise and form oligomers with other DJ-1 molecules, but the functional consequence of this phenomenon has been speculative (11,35,36,39,40). We show that in both control brain and SH-SY5Y cells, HMW DJ-1 complexes (240–480 kDa) are the predominant form of the protein, with some dimer present. HMW DJ-1 complex formation appears to be dependent on DJ-1 levels, since we were unable to detect formation of HMW complexes when expressing the intrinsically unstable variant DJ-1 L166P.

In human brain the levels of HMW DJ-1 complexes were unaffected in the substantia nigra and amygdala of PD brain, while it was increased in putamen. To our knowledge, no other studies have reported DJ-1 complexes in sporadic PD brains. Further work is required to investigate the role of oxidised DJ-1 in HMW complexes in brain, but also the relevance of the dimeric form.

Treatment induced oxidative stress or the expression of oxidation mimicking DJ-1 C106 DD and western blotting suggested oxidation dependent re-organisation of DJ-1 HMW complexes. Proteomic analysis of HMW complexes revealed the presence of proteins associated with RNA metabolism, which changed with the oxidation status of DJ-1. In line with this observation, DJ-1 has been shown to directly bind to mRNA species (17,41), and to be the regulatory subunit of a 400 kDa RNA binding complex (29), while SILAC proteomic analysis predicted that 11% of DJ-1 interactors are RNA binding proteins such as members of the hnRNP protein family (30), which we also found to immunoprecipitate with DJ-1. PABP1 was also found to be decreased in cytosolic C106DD DJ-1 complexes. This RNA-binding protein has been shown to relocate from the cytoplasm to the nucleus following treatment with hydrogen peroxide (42). Taken together, our data further support an oxidation dependent regulatory function of DJ-1.

Oxidation of DJ-1 at C106 perhaps in combination with additional redox-sensitive residues (cysteines C46, C53 and methionine residues) has been proposed to act as a regulatory switch for DJ-1 (8). So far four different oxidation levels for C106 have been reported, Cys-SH, Cys-SOH, Cys-SO_2_H, Cys-SO_3_H (23). Oxidation of C106 has been shown to be required for protection against mitochondrial dysfunction (7), oxidative stress and apoptosis via ASK1 (12). However, reports have suggested that the degree of oxidation may affect DJ-1 function. While mild oxidative stress allows DJ-1 to interact with ASK1 and exert protective actions, excessive oxidative stress attenuates this interaction and induces apoptosis (43). Furthermore, the level of -SO_2_H and -SO_3_H oxidation of Cys106 can perturb DJ-1 transcriptional function, with C106DD perhaps mimicking –SO_2_H oxidation (18,32). This might explain the differences in complex size, RNA transcriptome and catecholamine changes between oxidised endogenous or WT-DJ-1 complexes and complexes containing the oxidation mimic C106DD that we observed. The different complexes observed in human brain could reflect the different oxidation states of DJ-1 within these complexes or they could represent cell type specific forms.

The loss of DJ-1 complexes in KD1 cells might result in the uncontrolled activity of RNA associated proteins normally part of these complexes. Subsequent alterations in the transcriptome could therefore trigger changes in the amount or activity of catecholamine synthesising enzymes such as DBH and thereby cause impaired regulation of catecholamine synthesis, storage and/or release.

### DJ-1 and catecholamines

Our studies have indicated that oxidation or loss of DJ-1 complexes affected the catecholaminergic system at the transcriptomic level (VMAT1, ASK1, DDC and DBH) and by increasing intracellular levels of DA and NOR. The increased DA and NOR levels in DJ-1 KD cells were rescued by re-introduction of either WT or C106DD DJ-1 further demonstrating the importance of DJ-1 complexes in catecholaminergic homeostasis. Oxidation of endogenous DJ-1 or WT-DJ-1 could also increase DA and NOR levels suggesting that oxidation may fine-tune complex formation. However, since DD acted the same as WT DJ-1, this suggests that this oxidation mimic may act differently to complexes following induced oxidative stress, further supported by the differences observed in complex migration by BN-PAGE.

It should be noted that steady-state levels of DA, NOR and AD were measured by ELISA. Therefore, any changes in values may not only reflect changes in synthesis, but also the sequestration, release or turnover of these neurotransmitters: any of which could be affected by the presence or absence of DJ-1, or its oxidation status.

In accordance with our findings, changes at the transcriptome or protein level of the catecholamine system have been reported in relation with DJ-1 deficiency. Upon DJ-1 KO, DA transporter localisation to synaptic membranes increased, while protein levels were unaffected (44,45). Also, neurotransmitter receptor densities and levels have been reported to be affected by loss-of-DJ-1 (21,44). Altered DBH mRNA levels as well as total amounts of DBH protein have been observed in PD patients (46,47), although this is more likely to be reflective of catecholaminergic neuronal loss in substantia nigra and locus coeruleus, rather than changes in metabolism. Increased DBH activity has been reported in the medulla oblongata of PD patients (5). Conversely, polymorphisms decreasing DBH enzyme activity have been reported to be protective against PD (48,49).

In conclusion, our data suggest that DJ-1 function is required for catecholamine homeostasis. Fine-tuning of the underlying mechanism most likely occurs in an oxidation dependent manner, and seems to be mediated via DJ-1 HMW complexes which regulate the availability of RNA associated proteins. Upon KD of DJ-1 this regulatory mechanism is lost in neuroblastoma cells, suggesting that the lack of DJ-1 complexes may contribute to the increased susceptibility of DA and NOR neurons to neurodegeneration in PARK7-related PD. Since we observed decreased levels of oxidized DJ-1 in sporadic PD brains, this may affect catecholamine homeostasis in sporadic forms of PD.

## Materials and Methods

### Human post-mortem brain material

Substantia nigra (*n = *8) and amygdala (*n = *5) from control and PD brains were obtained from the Navarra Brain Bank (Pamplona, Spain). Frontal cortex was from the Netherlands Brain Bank. All PD cases met the UK Brain Bank Clinical Criteria for Parkinson’s disease. None of the cases presented dementia. All samples were used with the consent of the local ethics committee. Control and PD samples were age matched (control substantia nigra, mean age 68.5 ± 4.8 years; post mortem delay, 19.6 ± 5.2 h; PD substantia nigra, mean age 79.8 ± 2.9 years; post mortem delay, 19.2 ± 3.8 h; control amygdala, mean age 70.2 ± 3.1 years; post-mortem delay, 4.8 ± 1.0 h; PD amygdala, mean age 76.2 ± 1.6 years; post mortem delay, 5.4 ± 1.3 h). Samples were assessed to have Braak staging 3–6. The cortex samples were from 10 agematched controls (*n = *10, mean age 78.4 ± 2.6 years, post mortem delay 6.7 ± 0.5 h) and PD patients (*n = *10, mean age 71.9 ± 2.0 years, post mortem delay 6.4 ± 0.5 h).

### Cultured cell lines

The human neuroblastoma SH-SY5Y cell line was obtained from the European Collection of Cell Cultures. SH-SY5Y cell lines were cultured in DMEM/F-12 Nutrient Mix (1:1) with GlutaMAX^TM^-I (Invitrogen) supplemented with 10% (v/v) foetal calf serum (BioSera), penicillin (50 U/ml final), streptomycin (50 ng/ml), sodium pyruvate (1 mM) and non-essential amino acids. To generate DJ-1 KD cells, SH-SY5Y cells were transfected with pGIPZ lentiviral shRNA plasmid (clone ID: V2LHS_207558; Thermo Scientific) encoding siRNA against the 3’UTR of DJ-1 (Target sequence: CCTACAAATTGTGTCTATA). Two DJ-1KD lines (KD1 and KD2) and a scrambled control cell line (SC1) were used in this study.

For some experiments KD1 cells were transiently transfected with the respective DNA constructs (WT DJ-1, oxidation deficient mutant DJ-1 C106A or oxidation mimicking artificial mutation DJ-1 C106DD in mammalian expression vector pCDNA4; DJ-1 WT or DJ-1 L166P in mammalian expression vector pCDNA3.1) using xtreme GENE HP DNA transfection reagent (Sigma) as previously described (50). Briefly, 2 × 10^5^ cells/ml were seeded in complete culture medium. Cells were transfected with a mix of 2 µg plasmid DNA and 2 µl of transfection reagent. Cells were analysed 72 h after transfection. Stable cell lines expressing DJ-1 WT or DJ-1 C106DD in KD1 cells were generated using Superfect TR (Qiagen). Briefly, cells were seeded onto an 8 cm culture dish. When the cells reached 50% confluency they were transfected with a mix of 4 µg plasmid DNA and 4 µl of transfection reagent. Selection with respective antibiotic phleomycin (zeocin) was started 48 h later and selection continued for 6–8 weeks. Colonies were harvested and cultured individually and positive clones were analysed for the presence of DJ-1 by western blot.

### Stress treatment to induce intracellular oxidative stress

To induce intracellular oxidative stress, cells were pre-treated with 1 mM buthionine sulfoximine (BSO) for 24 h to reduce glutathione levels (51) and subsequently treated with 1 mM H_2_O_2_ for 1 h. No significant cell death was observed. For stress treatments prior to qPCR analysis or ELISA, cells were left for an additional hour in fresh media to recover.

### Homogenisation of post-mortem brain tissue

For SDS PAGE analysis brain tissue was homogenised (10 mg/100 μl buffer) in 10 mM Tris, pH7.4, 150 mM NaCl, 0.1% (w/v) SDS, DNase (50 Units; Promega), 1X DNase buffer and protease inhibitors using a plastic homogeniser in 1.5 mL reaction tubes. Samples were then homogenised at 1000 rpm until homogenous. Samples were then incubated at 37 °C for 1 h to digest DNA.

### Polyacrylamide gel electrophoresis

Blue native polyacrylamide gel electrophoresis (BN PAGE) was performed as described by Schägger *et al.* (52). Protein extraction was performed by sonication of brain/SH-SY5Y cell pellets in sucrose buffer (250 mM Sucrose, 10 mM HEPES, 1.5 mM MgCl_2_, 10 mM KCl, pH 7.0) applying pulses of 5 s (cells: 1 pulse at 50% intensity and 3 pulses at 30% intensity; brain: 2 pulses at 50% intensity and 3 pulses at 30% intensity) with breaks of 30 s on ice. Protein (25–80 µg) was separated on 7.5% fixed percentage gels or gradient gels (8–16%) when resolving monomeric, dimeric and complex bound DJ-1. In order to denature HMW complexes and to facilitate protein transfer to Hybond P membrane (GE Healthcare), gels were incubated in solubilisation buffer (2% (w/v) SDS, 66 mM Sodium hydrogen carbonate, 2% (v/v) β-mercaptoethanol, 6 M urea) for 20 min on a shaker and subsequently washed in transfer buffer (20% (v/v) methanol, 25 mM Tris pH 8.8, 0.2 M glycine) for 10 min prior to transfer.

SDS PAGE was performed using NuPAGE^®^ 12% or NuPAGE^®^ 4–12% gels (Invitrogen). SH-SY5Y cells were harvested with trypsin and lysed on ice in 0.1–0.2% (v/v) SDS in Tris-HCl, pH 7.4 supplemented with protease inhibitor cocktail (Pierce) and Benzonase (Merck Millipore). Protein samples (50 µg) were mixed with SDS sample loading buffer and 1X reducing agent (both Invitrogen) and heated to 70 °C for 5 min. An aliquot of protein samples prepared for BN-PAGE above were denatured and heated in a similar fashion to check for β-actin levels as a further loading control.

For two-dimensional BN/SDS PAGE, samples were separated by BN PAGE as above except 50 µg protein was used. Gel lanes of interest were cut out, incubated in solubilisation buffer (2% (w/v) SDS, 66 mM Sodium hydrogen carbonate, 2% (v/v) β-mercaptoethanol) for 10 min at room temperature (RT) on a shaker and subsequently washed in equilibration buffer (50 mM Tris, 1% (w/v) SDS) for 2 × 10 min at RT on a horizontal shaker. Pre-treated gel strips were placed between Mini-Protean^®^ 0.75 mm glass plates (BioRad) and cast in 4% polyacrylamide-SDS-urea stacking gel. Protein was then separated in the second dimension using a 12.5% polyacrylamide-SDS-urea resolving gel.

### Western blotting analysis

Separated proteins were transferred to Hybond P membrane (GE Healthcare). BN PAGE membranes were destained with methanol and stained with Ponceau to mark protein ladder (Native Mark Unstained Protein Standard, Life Technologies) and check for equal protein loading. Membranes were blocked for 30 min at room temperature in 10% milk in PBS and subsequently probed with monoclonal mouse anti-total DJ-1 (3E8, Enzo Life Sciences; 1:5,000), monoclonal mouse anti-oxidised DJ-1 (clone M106, kind gift from Yoshiro Saito; 1:1,000), anti-hnRNPA1 (abcam ab50966; 1:1000), anti-GAPDH (abcam ab8245; 1:40,000), anti-hsc70 (abcam ab51052), anti-PABP1 (abcam ab21060; 1:50) and anti-β-actin (abcam ab82618; 1:50,000). After washing with PBS/0.4% (v/v) Tween20 (PBS/T), blots were incubated with respective horse radish peroxidase-coupled secondary antibodies (Dako). Blots were developed with either in-house chemiluminescence solution or enhanced chemiluminescence kit (Pierce). Densitometry of bands was quantified using imageJ (NIH).

### Co-immunoprecipitation of HMW DJ-1 complexes

Co-immunoprecipitation was performed using the Novex Dynabeads^®^ system (Invitrogen). First, polyclonal goat anti DJ-1 antibody (ab4150; abcam) was covalently linked to Dynabeads^®^ M-270 Epoxy beads using the Dynabeads^®^ Antibody Coupling Kit (Invitrogen) following the manufacturer’s instructions. Typically 10 µg of antibody was used per 1 mg of Dynabeads^®^ M-270 Epoxy. For the co-immunoprecipitation reaction, six confluent 10 cm tissue culture dishes of SH-SY5Y cells were harvested by trypsination and washed in PBS. Cells were lysed in 50 mM Tris, 0.27 M sucrose, 1 M sodium orthovanadate, 1 mM EDTA, 1 mM EGTA, 10 mM β-glycerophosphate, 5 mM pyrophosphate, 50 mM sodium fluoride, 1% NP40, 150 mM sodium chloride; pH 7.5 and cell lysates were concentrated to a volume of 500 µl using Amicon^®^ Ultra Centrifugal Filters Ultracel^®^ -100 K (Milipore) in order to remove proteins less than 100 kDa, including monomeric and dimeric DJ-1. Lysate was then incubated with the antibody-bound beads for 1 h at 4 °C. The beads were then washed twice with PBS and stored in PBS at 4 °C until subsequent mass spectrometric analysis was performed.

### Label-free quantitative liquid chromatography tandem mass spectrometry (LC-MS/MS)

LC-MS/MS analysis was performed at the Biological Mass Spectrometry Facility at the Institute of Child Health, University College London, UK. Briefly, samples were reduced, alkylated and subjected to trypsination over night at 37 °C. After digestion, 10 μl of neat supernatant (or 9 μl of supernatant and 1 μl of yeast enolase) was pipetted into a sample vial and loaded on to an autosampler for automated liquid chromatography (LC) tandem mass spectrometry (MS/MS) analysis. All LC–MS/MS experiments were performed using the nanoAcquity UPLC and QTOF Premier mass spectrometer (Waters Corporation, Manchester, UK). After the run, data were processed using ProteinLynx Global server 2.5 (PLGS) software. All samples were analysed in quadruplicates. Quantification data were obtained at least in a triplicate; ID data in a quadruplicate. Quantification data were obtained as recently described by Manwaring *et al.* (37).

### Quantitative real-time PCR

RNA was extracted from cells with RNeasy mini kit (Qiagen) and 500 ng RNA converted to cDNA with reverse transcriptase (Primerdesign). DJ-1 mRNA levels were measured with Power SYBRgreen and a STEP One PCR machine (Applied Biosystems). Data were normalised against β-actin by the ΔCT method (DJ-1 primer sequences: forward, 5’-TGGCTGGAAAAGACCCAGTA-3, reverse, 5’-CCTTCACAGCAGCAGACTCA-3’; β-actin, forward, 5’-TCTACAATGAGCTGCGTGTG-3’, reverse, 5’-GGTGAGGATCTTCATGAG GT-3’VMAT1 forward, 5’-GGTGGATTCTTCTATGATGCCC-3’, reverse, 5’-GTGGATGGACCTATAGCAAAG-3’, DBH forward, 5’-GGCCGGGAGTGGGAGATCGT-3’, reverse, 5’-TGTGGCCAGCTCCCGGTCTT-3’ and ASK1 forward, 5’-AGACATCTGGTCTCTGGGCTGTAC-3’, reverse, 5’-AACATTCCCACCTTGAACATAGC-3’)

### RNA sequencing

RNA and small RNA sequencing experiments were performed as previously described (53,54). In brief, RNA-seq and small RNA-seq libraries were prepared using the TruSeq^®^ RNA Sample Preparation v2 kit and the TruSeq^®^ Small RNA Preparation kit from Illumina according to the manufacturer’s instructions. The library quality was assessed using an Agilent 2100 Bioanalyzer. Sample concentrations were measured using a Qubit dsDNA HS Assay Kit and adjusted to 2 nM before sequencing (50 bp single end) on a HiSeq 2000 instrument (Illumina) using TruSeq SR Cluster Kit v3-cBot-HS and TruSeq SBS Kit v3-HS according to the manufacturer’s instructions.

RNA-sequencing data were analyzed using in-house workflows and resulted in 17–32 million reads per sample (Supplementary Material, Table S3). Quality assessment was based on the raw reads using the FASTQC quality control tool (v0.10.1). The sequence reads (single-end 50 bp) were aligned to the human reference genome (hg19) with STAR aligner (2.3.0e_r291). Read counts for all genes and all exons (Ensembl annotation v72) were obtained using FeaturesCount (version 1.4.6). Differential expression analysis was carried out using gene read counts with DESeq2 package (v1.4.5) (55). Genes with less than 10 reads (baseMean) were filtered out. Genes with a p value ≤ 0.05 and log2 fold change > 0.5 were considered to be differentially expressed. Small RNA-sequencing data were analyzed with the Oasis sRNA detection and differential expression modules using standard parameters and resulted in 13.78 x 10^6^ and 12.45 x 10^6^ reads per sample for WT and DD samples, respectively (56).

To identify enriched GO categories, the web-service WebGestalt was used. GO category enrichment was assessed by calculating the fold-change between observed and expected number of genes of a given GO category, where terms were scored enriched if they had an adjusted P value < 0.05. In addition, we used QIAGEN’s Ingenuity^®^ Pathway Analysis (IPA^®^; www.qiagen.com/ingenuity) to obtain RNA-miRNA correlation and pathway information.

### Catecholamine ELISA to measure intracellular levels of DA, noradrenaline and adrenaline

To measure catecholamine levels in the different SH-SY5Y cell lines TriCat ELISA from IBL International (Germany) was used according to the manufacturer’s instructions. Briefly, around 100 × 10^6^ cells were harvested in Versene (Invitrogen) supplemented with 0.125% (v/v) Trypsin (Invitrogen), washed in PBS, and pelleted at 400 × g for 10 min. The pellet was subsequently resuspended in 100 µl ddH_2_O, sonicated with two pulses at 50% intensity for 5 s and cooled on ice for 30 s in between. The lysate was then made up to 500 µl with ddH_2_O and used to extract catecholamines in extraction buffer as supplied by the manufacturer. Values were normalised against protein concentration of the samples.

## Supplementary Material

Supplementary Material is available at *HMG* online.

## Supplementary Material

Supplementary Figures and Tables
