# DJ-1 is a redox sensitive adapter protein for high molecular weight complexes involved in regulation of catecholamine homeostasis

**DOI:** 10.1093/hmg/ddx425

**Published:** 2017-12-21

**Authors:** Dominik Piston, Lydia Alvarez-Erviti, Vikas Bansal, Daniela Gargano, Zhi Yao, Gyorgy Szabadkai, Mark Odell, M Rhyan Puno, Benny Björkblom, Jodi Maple-Grødem, Peter Breuer, Oliver Kaut, Jan Petter Larsen, Stefan Bonn, Simon Geir Møller, Ullrich Wüllner, Anthony H V Schapira, Matthew E Gegg

**Affiliations:** 1 Department of Clinical Neuroscience, UCL Institute of Neurology, London, UK; 2 Norwegian Centre for Movement Disorders, Stavanger University Hospital, Stavanger, Norway; 3 German Centre for Neurodegenerative Diseases (DZNE), Bonn, Germany; 4 Molecular Neurobiology, Centre for Biomedical Research of Rioja, Logrono, Spain; 5 Computational Systems Biology, Site Göttingen German Center for Neurodegenerative Diseases (DZNE), Göttingen, Germany; 6 Centre for Organelle Research, University of Stavanger, Stavanger, Norway; 7 Department of Cell and Developmental Biology, Consortium for Mitochondrial Research, University College London, London, UK; 8 Department of Molecular and Applied Biosciences, University of Westminster, London, UK; 9 Department of Chemistry, Umeå University, SE-90187 Umeå, Sweden; 10 Department of Neurology, University of Bonn Medical Centre, Bonn, Germany; 11 Department of Biological Sciences, St. John's University, New York, NY, USA

*Human Molecular Genetics*, 2017, 26(20), 4028-4041. doi: 10.1093/hmg/ddx294 The authors wish to apologize for an error in the reported affiliation of Vikas Bansal and Stefan Bonn, who were originally listed under the affiliation ‘German Centre for Neurodegenerative Diseases (DZNE), Bonn, Germany’ rather than ‘Computational Systems Biology, Site Göttingen German Center for Neurodegenerative Diseases (DZNE), Göttingen, Germany’. This error has now been corrected online.

