# Supplementary material for: DJ-1 is a redox sensitive adapter protein for high molecular weight complexes involved in regulation of catecholamine homeostasis
Source: Hum Mol Genet. 2017 Jul 25;26(20):4028–41. doi: 10.1093/hmg/ddx294 (PMC5886150; doi:10.1093/hmg/ddx294)
Supplement: Supplementary Figures and Tables [file supplementary_data_ddx294.pdf]

Supplementary Figure 1

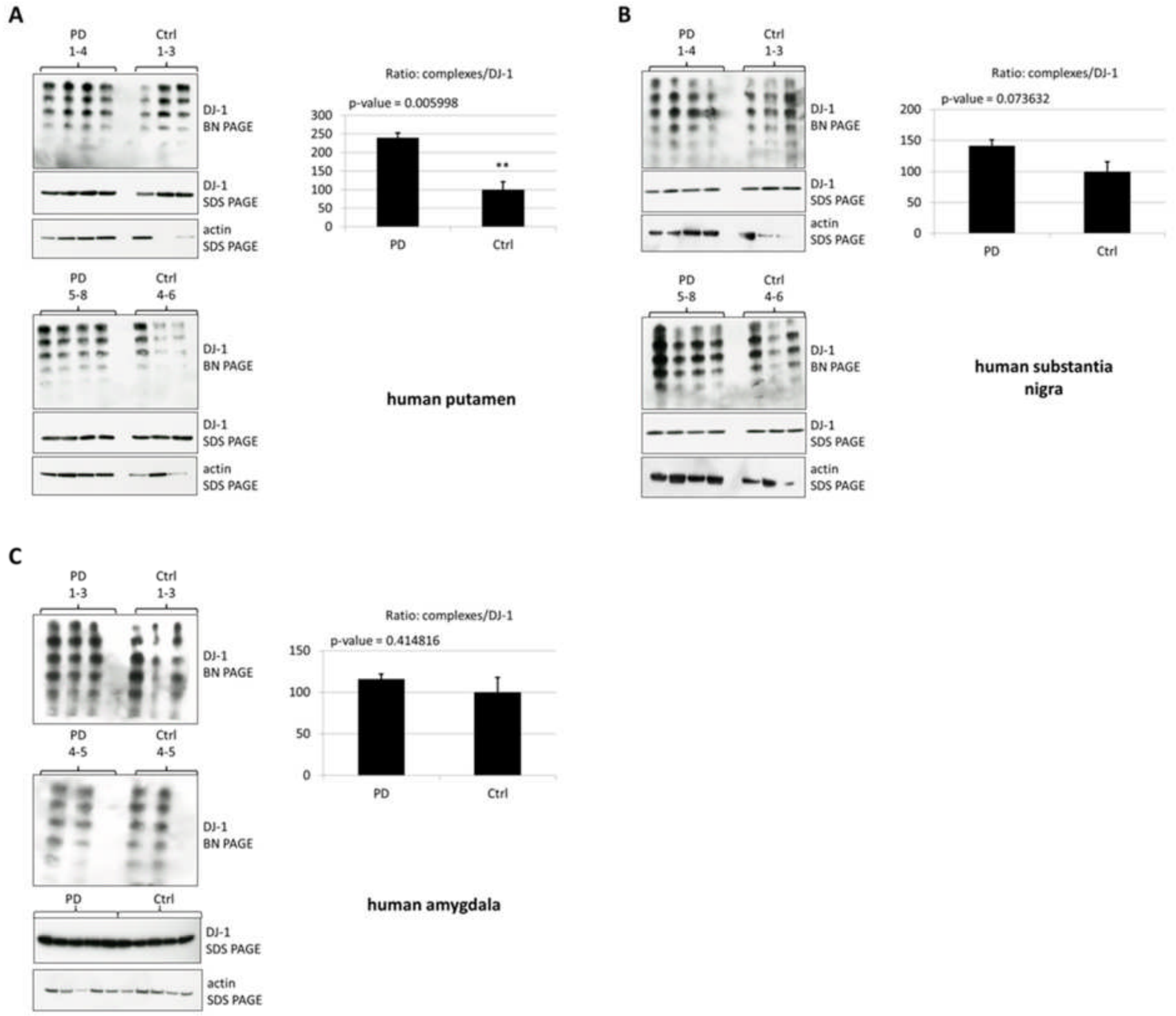

Supplementary Fig. 1. Total levels of DJ-1 complexes in human post mortem brain tissue. Soluble HMW complexes were extracted from putamen, substantia nigra and amygdala from frozen human post mortem brain tissue of controls and PD patients and analysed by BN PAGE and western blotting for total DJ-1. The same amount of each sample was also analysed by SDS PAGE to measure total DJ-1 levels and β-actin. Total levels of DJ-1 complexes were quantified by measuring density of all complexes and normalised to total levels of DJ-1 detected by SDS PAGE. **A**) Respective western blots and quantification of human putamen samples (**p = 0.007). **B**) Respective western blots and quantification of substantia nigra samples (p = 0.07, not significant). **C**) Respective western blots and quantification of human amygdala samples (p = 0.41, not significant). For amygdala SDS-PAGE analysis, all samples analysed on one blot. Sample order: PD 1-5, Ctrl 1-5.

Supplementary Figure 2

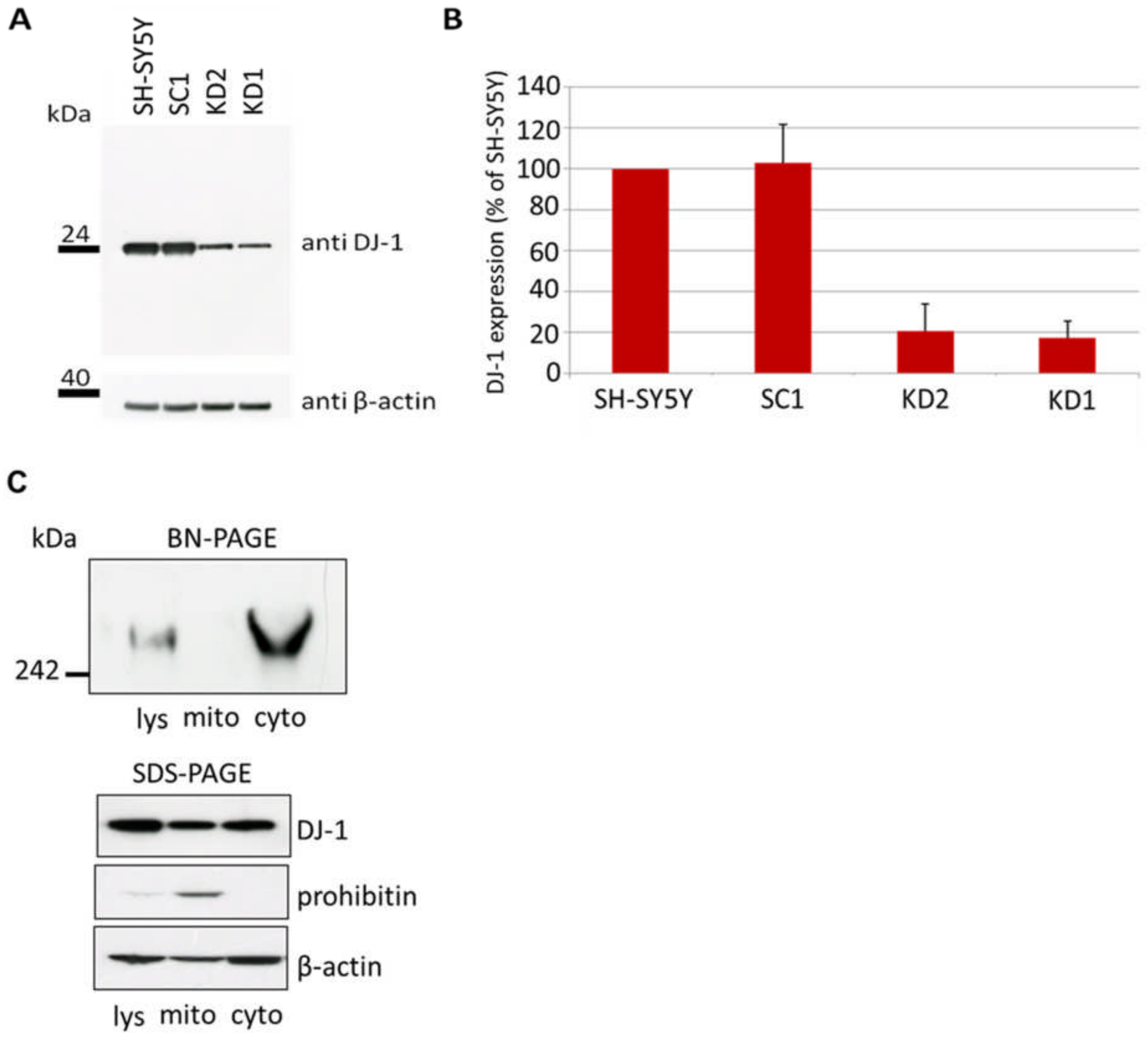

Supplementary Fig. 2. Characterisation of the scrambled control cell line SC1 and the DJ-1 knock down cell lines KD2 and KD1. A) DJ-1 protein levels were analysed by SDS PAGE and western blotting analysis using the monoclonal DJ-1 antibody. β-actin was used as loading control. A representative western blot is shown (n = 3). **B)** Quantification of the DJ-1 protein levels in the different cell lines as percentage of SH-SY5Y cells. DJ-1 levels were unaffected in SC1 cells, whereas they were decreased by 79.3% and 82.8% in KD2 and KD1 cells, respectively (n=3). **C) Upper panel:** Crude subcellular fractionation was performed on SH-SY5Y cells. Total lysates, mitochondrial and cytosolic fractions were separated by BN PAGE and western blot analysis for DJ-1 was performed. The HMW complex was detectable in the total lysates and cytosolic fractions, but not mitochondrial fractions. **Lower panel:** The same fractions were also analysed by SDS PAGE and subsequent western blotting to measure total DJ-1 levels in the different fractions. DJ-1 was detected in both mitochondrial and cytosolic fractions. The membranes were also probed with antibodies against the mitochondrial marker prohibitin and β-actin as a cytosolic marker, to check the purity of the fractions.

Supplementary Figure 3

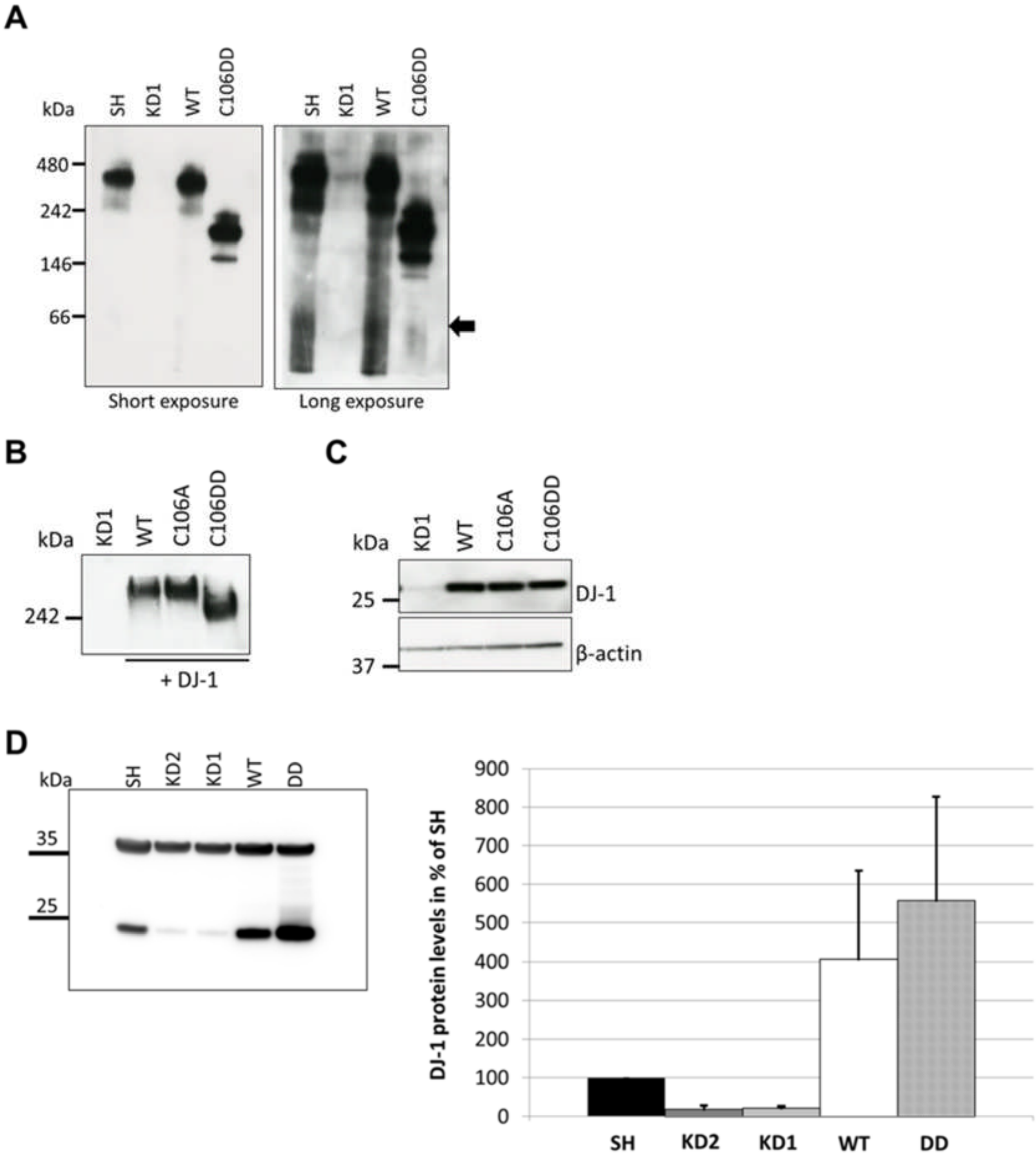

Supplementary Fig. 3. DJ-1 complexes are the predominant species in SH SY5Y cells. A) Soluble protein complexes were extracted from SH-SY5Y cells, KD1 cells and KD1 cells transiently transfected with DJ-1 WT or C106DD 72 h after transfection, separated on 8-16% gradient BisTris gels and western blot analysis was performed. With longer exposure times more than two DJ-1 positive complexes were detected and potentially dimeric DJ-1 (arrow). **B)** Soluble protein complexes were extracted from KD1 and KD1 cells transiently transfected with DJ-1 WT, C106A or C106DD 72 h after transfection, separated on 7.5% BisTris gels and western blot analysis was performed. Reintroduction of all three DJ-1 variants rescued complex formation in the knock down cell line. However, while expression of DJ-1 WT and C106A resulted in the formation of a HMW complex with similar size as observed before (SH-SY5Y and SC1 cells; Fig. 3), the DJ-1 complex was shifted to a lower molecular weight upon expression of oxidation mimic DJ-1 C106DD. **C)** The same volume of each sample was also analysed by SDS PAGE and western blotting, probing for DJ-1 and actin in order to confirm

equal protein expression levels. **D)** SDS page and western blotting for DJ-1 in SH, KD1, KD2 and KD1 cells with stable expression of WT and C106DD DJ-1. DJ-1 protein expression was normalised against β-actin and expressed as percent of SH-SY5Y cells in the bar graph (n=3). DJ-1 protein levels in KD1 and KD2 cells are 21% and 19%, respectively. DJ-1 levels are 406% in WT cells and 558% in DD cells.

Supplementary Figure 4

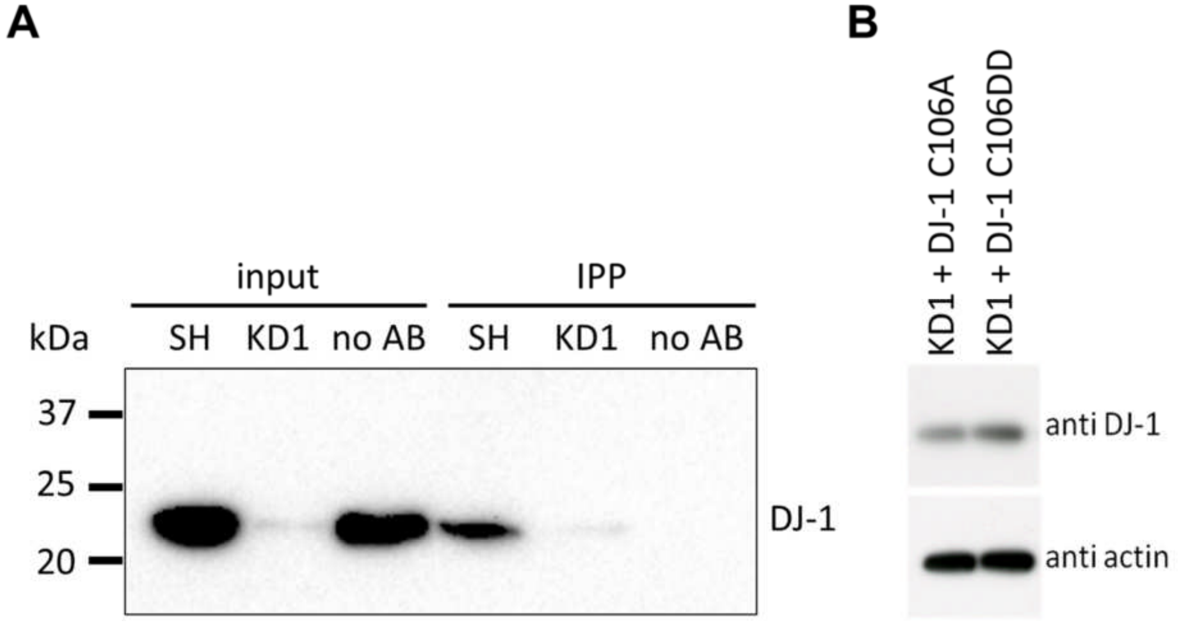

Supplementary Fig. 4. Immunoprecipitation of DJ-1 from SH-SY5Y cells and confirmation of equal DJ-1 expression levels prior to mass spectrometry analysis. A) SH-SY5Y (SH) or DJ-1 KD1 cells were lysed and DJ-1 immunoprecipitated with polyclonal goat antibody against DJ-1. Following release from beads, pulled down protein was separated by SDS-PAGE and a western blot performed using mouse monoclonal DJ-1 antibody. DJ-1 was present in SH and no antibody (no ab) lysates (input). DJ-1 was immunoprecipitated from SH-SY5Y cells, but as expected very little in DJ-1 KD1 cells and absent in the no ab control. **B)** An aliquot of the cell lysates used to immunoprecipitate HMW DJ-1 complexes from KD1 cells transfected with DJ-1 C106A or C106DD and then analysed by LC-MS/MS was reserved to measure DJ-1 protein levels by western blot. Beta actin was used as a loading control. DJ-1 expression levels were similar in both samples.

Supplementary Figure 5

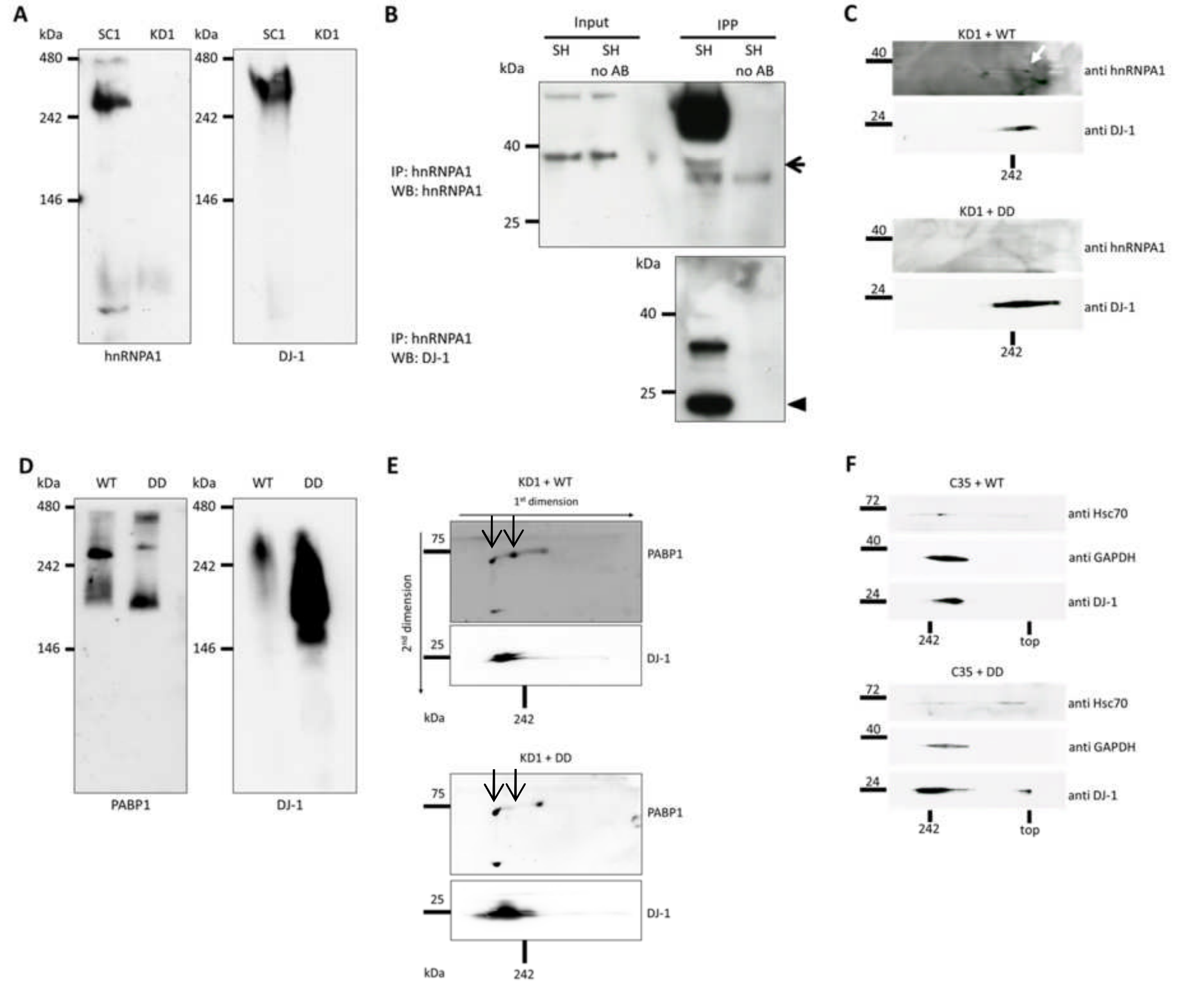

Supplementary Fig. 5. Confirmation of DJ-1 complex components as identified by mass spectrometry. A) BN PAGE and subsequent western blotting probing for DJ-1 and hnRNPA1 suggest that both DJ-1 and hnRNPA1 are components of the same HMW complex. Both proteins are detectable in SC1 cells, but not in KD1 cells and the signals appear at the same molecular weight. **B)** Immunoprecipitation experiments confirmed the interaction of DJ-1 and hnRNPA1. hnRNPA1 antibody pulled down DJ-1 from normal SH cells. Both DJ-1 (arrow head) and hnRNPA1 (arrow) were detected after immunoprecipitation at the expected molecular weight, but not in no antibody control (no AB). **C)** Two dimensional BN/SDS PAGE and western blotting detected both DJ-1 and hnRNPA1 (white arrow) at the correct molecular weight. Their similar positions in 2D gels suggest they are components of the same HMW complex. **D)** BN PAGE and western blotting probing for DJ-1 and PABP1 suggest the two proteins are components of the same HMW complex in WT cells and DD cells. As expected PABP1 expression has a lower molecular weight in DD cells since DJ-1 complexes have a lower apparent molecular weight in these cells. **E)** Two dimensional BN/SDS PAGE of WT cells yielded two spots of the expected size for PABP1 (arrows) that co-migrated with DJ-1. One of these dots was absent in C106DD DJ-1 HMW complexes. This suggests that DJ-1 and PABP1 are components of the same complex in WT cells, but that there is a different

composition in DD cells. **F)** Two dimensional BN/SDS PAGE also confirmed Hsc70 and GAPDH as potential DJ-1 complex components.

Supplementary Figure 6

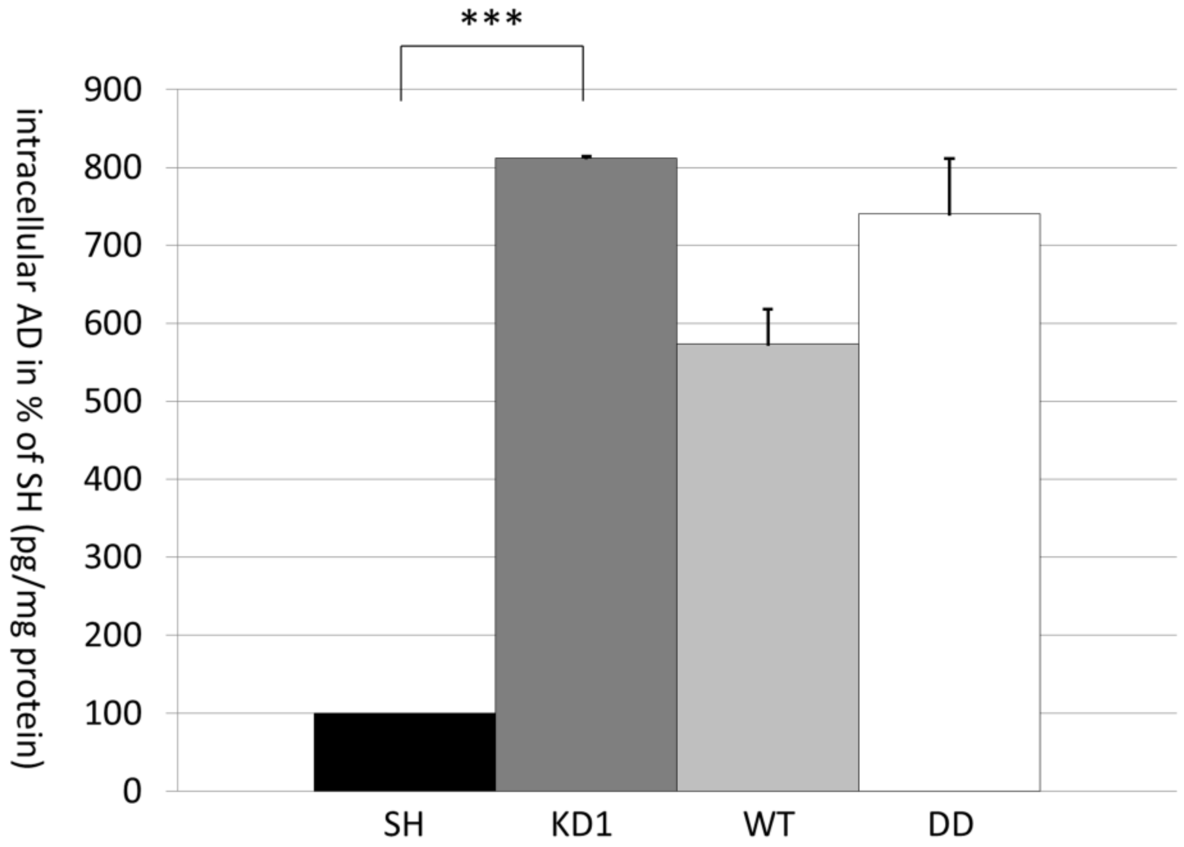

Supplementary Fig. 6. Adrenaline levels increased in KD1 cells and cells expressing WT or C106DD. Intracellular levels of adrenaline (AD) were measured by ELISA and calculated as pg AD/mg protein. Results are expressed as percent of SH-SY5Y cells (n = 3; measured three times in three biological replicates). AD levels in KD1 cells were significantly increased 3.7 fold, 9.8 fold and **8.1 fold** when compared to normal SH-SY5Y cells (median = 7.2 fold). AD levels in cells expressing DJ-1 WT were increased (0.3 fold, 1.9 fold, **5.7 fold**) when compared to normal SH-SY5Y cells (median = 2.6 fold). AD levels in cells expressing DJ-1 DD were increased (1.5 fold, 8.9 fold, **7.4 fold**) when compared to normal SH-SY5Y cells (median = 5.9 fold). Fold changes in bold are from representative experiment shown in the graph. *** P < 0.001 versus SH.

Supplementary Figure 7

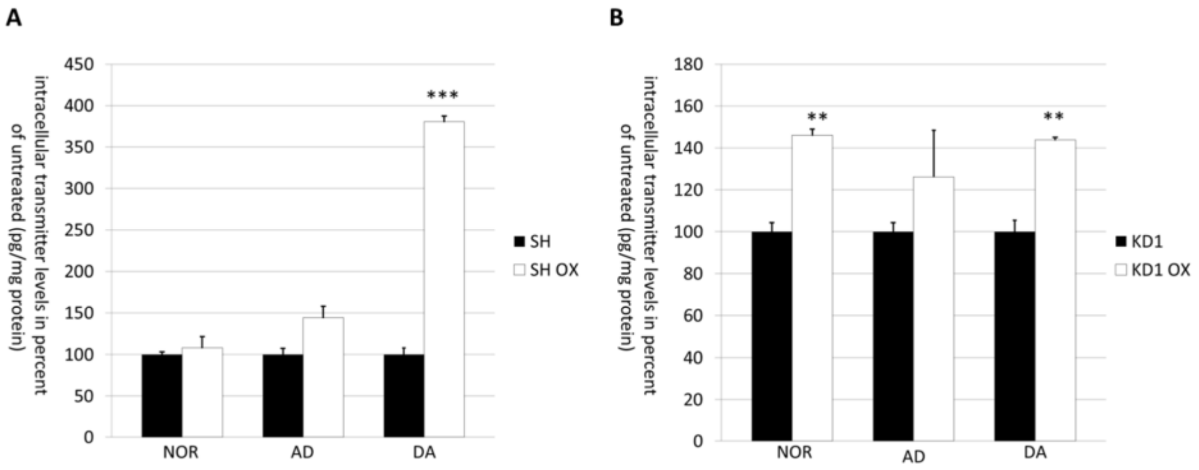

Supplementary Fig. 7 Catecholamine ELISA to measure intracellular levels of dopamine (DA), noradrenaline (NOR), and adrenaline (AD) under oxidizing conditions SH-SY5Y cells were treated with (1mM BSO for 24 h followed by 1 mM H_2O_2 for 30 min) and catecholamine levels were calculated (pg/mg protein). Results are expressed as percent of untreated cells (black bars; measured once in three biological replicates). **A)** In SH-SY5Y cells levels of NOR and AD were not affected by stress treatment, whereas levels of DA were significantly increased after treatment (p = 0.0005). **B)** In KD1 cells levels, NOR and AD were significantly increased after stress treatment (p-value for NOR = 0.005; p-value for DA = 0.001).

Supplementary Fig. 8. RNA Seq: Data assessment for GO terms

A) Downregulated mRNA targets in WT versus DD

p-value ≤ 0.05 and log2 fold change > 0.5

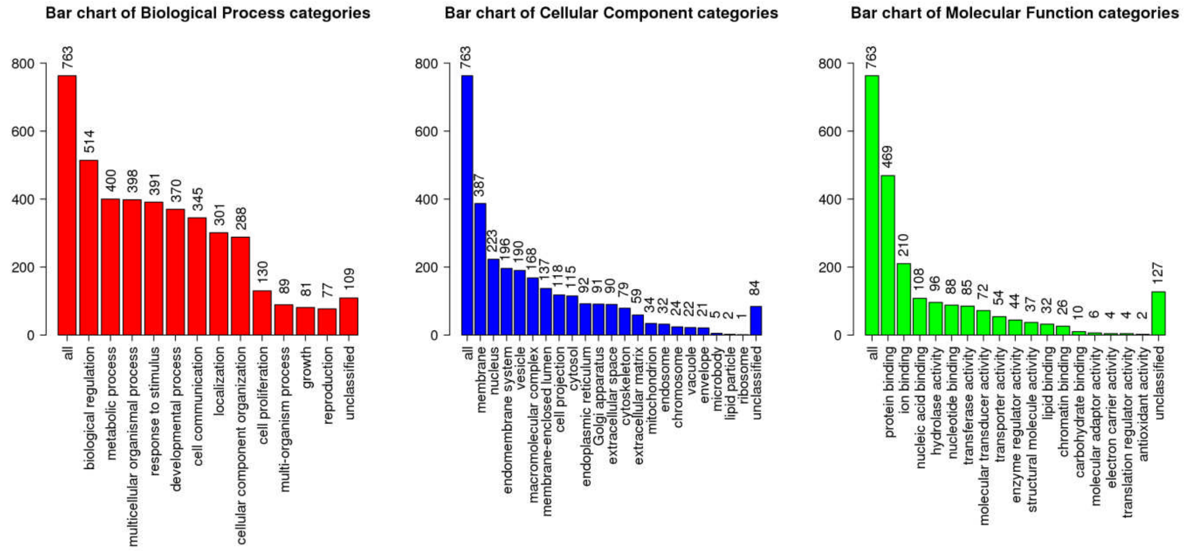

B) Upregulated mRNA targets in WT versus DD

p-value ≤ 0.05 and log2 fold change > 0.5

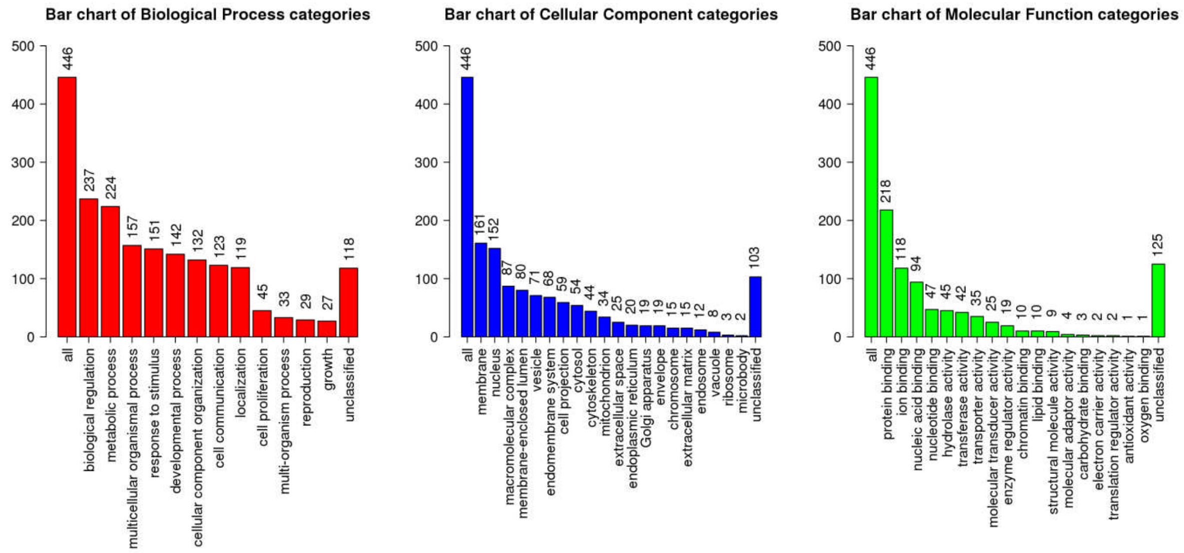

Supplementary Table 1. Mass spectrometric analysis of HMW DJ-1 complexes in SH-SY5Y cells. No = Number; Accession and Entry codes for Uniprot; Description of the identified protein; theoretical values for molecular weight (mW) in Daltons (Da) and isoelectric point (pI) in pH units; mean amount in femtomoles (fmol) based on MS Label-free quantification.

No	Accession	Entry	Description	mW (Da)	pI (pH)	Mean (fmol)
1	ACTG_HUMAN	P63261	Actin cytoplasmic 2	41765	5.1606	183.9541
2	PARK7_HUMAN	Q99497	Protein DJ 1	19878	6.3721	157.2935
3	ENO1_YEAST	P00924	Enolase 1	46787	6.1538	100
4	TRYP_PIG	P00761	Trypsin	24393	6.9141	77.49935
5	VIME_HUMAN	P08670	Vimentin	53619	4.8633	44.6505
6	CH60_HUMAN	P10809	60 kDa heat shock protein mitochondrial	61016	5.5503	42.9664
7	ACTB_HUMAN	P60709	Actin cytoplasmic 1	41709	5.1431	32.5744
8	EF1A3_HUMAN	Q5VTE0	Putative elongation factor 1 alpha like 3	50153	9.4131	32.2976
9	K2C1_HUMAN	P04264	Keratin type II cytoskeletal 1	65998	8.2661	24.9905
10	G3P_HUMAN	P04406	Glyceraldehyde 3 phosphate dehydrogenase	36030	8.6968	23.8184
11	ALBU_HUMAN	P02768	Serum albumin	69321	5.8608	22.70233
12	CG025_HUMAN	Q9BPX7	UPF0415 protein C7orf25	46422	5.9766	21.3645
13	K1C9_HUMAN	P35527	Keratin type I cytoskeletal 9	62026	4.9585	19.01508
14	K1C10_HUMAN	P13645	Keratin type I cytoskeletal 10	58791	4.9556	15.68173
15	TBA1A_HUMAN	Q71U36	Tubulin alpha 1A chain	50103	4.7622	14.2412
16	ZA2G_HUMAN	P25311	Zinc alpha 2 glycoprotein	34237	5.6367	10.965
17	SERPH_HUMAN	P50454	Serpin H1	46411	9.0439	10.07058
18	ROA1_HUMAN	P09651	Heterogeneous nuclear ribonucleoprotein A1	38723	9.3677	9.6231
19	HNRPC_HUMAN	P07910	Heterogeneous nuclear ribonucleoproteins C1 C2	33649	4.7593	8.910425

20	TBB5_HUMAN	P07437	Tubulin beta chain	49638	4.5908	8.746125
21	ERH_HUMAN	P84090	Enhancer of rudimentary homolog	12251	5.5342	6.123533
22	PHB_HUMAN	P35232	Prohibitin	29785	5.4302	5.8317
23	HBD_HUMAN	P02042	Hemoglobin subunit delta	16045	8.2397	5.6682
24	HBA_HUMAN	P69905	Hemoglobin subunit alpha	15247	9.1787	5.5543
25	SODC_HUMAN	P00441	Superoxide dismutase Cu Zn	15925	5.666	5.402
26	ROA2_HUMAN	P22626	Heterogeneous nuclear ribonucleoproteins A2 B1	37406	9.1948	5.3118
27	SREK1_HUMAN	Q8WXA9	Splicing regulatory glutamine lysine rich protein 1	59345	10.8721	5.2472
28	ACY2_HUMAN	P45381	Aspartoacylase	35712	6.0542	4.4187
29	K1C25_HUMAN	Q7Z3Z0	Keratin type I cytoskeletal 25	49287	4.812	2.4863
30	EF1A1_HUMAN	P68104	Elongation factor 1 alpha 1	50109	9.3428	2.2642
31	TBB2B_HUMAN	Q9BVA1	Tubulin beta 2B chain	49920	4.5908	1.968
32	OSTP_HUMAN	P10451	Osteopontin	35401	4.1777	1.3
33	TBB4B_HUMAN	P68371	Tubulin beta 4B chain	49799	4.6025	1.2753

Supplementary Table 2. Differences in HMW DJ-1 complex composition between cells expressing DJ-1 C106A and C106DD. No = Number; Accession and Entry codes for Uniprot; Description of the identified protein; theoretical values for molecular weight (mW) in Daltons (Da); mean amount in femtomoles (fmol) based on MS Label-free quantification; fold change based on alterations in mean amount between samples C106A and C106DD.

No	Accession	Entry	Description	mW (kDa)	Mean (fmol) in C106A	Mean (fmol) in C106DD	Fold change (C106DD/C106A)
1	RA1L2 HUMAN	Q32P51	Heterogeneous nuclear ribonucleoprotein A1 like 2	34.20	76.06	15.13	0.199
2	HSP72 HUMAN	P54652	Heat shock related 70 kDa protein 2	69.98	7.69	2.77	0.360
3	PABP1 HUMAN	P11940	Polyadenylate binding protein 1	70.63	5.65	2.59	0.459
4	ACTG HUMAN	P63261	Actin cytoplasmic 2	41.77	690.95	317.55	0.460
5	MYH10 HUMAN	P35580	Myosin 10	228.86	6.41	3.30	0.515
6	H2B1N HUMAN	Q99877	Histone H2B type 1 N	13.91	33.49	17.39	0.519
7	MYO1B HUMAN	O43795	Unconventional myosin Ib	131.90	18.27	9.53	0.522
8	ERH HUMAN	P84090	Enhancer of rudimentary homolog	12.25	9.50	4.99	0.526
9	RLA0 HUMAN	P05388	60S acidic ribosomal protein P0	34.25	7.13	3.75	0.526
10	DREB HUMAN	Q16643	Drebrin	71.39	38.28	20.18	0.527
11	CALM HUMAN	P62158	Calmodulin	16.83	62.83	33.62	0.535
12	MYH9 HUMAN	P35579	Myosin 9	226.39	16.35	8.98	0.549
13	ENOA HUMAN	P06733	Alpha enolase	47.14	9.81	5.40	0.550
14	H2B1M HUMAN	Q99879	Histone H2B type 1 M	13.98	33.32	18.47	0.554
15	HNRPL HUMAN	P14866	Heterogeneous nuclear ribonucleoprotein L	64.09	12.85	7.31	0.569
16	ROA1 HUMAN	P09651	Heterogeneous nuclear ribonucleoprotein A1	38.72	56.33	32.67	0.580
17	THOC4 HUMAN	Q86V81	THO complex subunit 4	26.87	7.19	4.18	0.581

18	H2A1 HUMAN	P0C0S8	Histone H2A type 1	14.08	46.66	27.47	0.589
19	MYL6 HUMAN	P60660	Myosin light polypeptide 6	16.92	11.91	7.07	0.594
20	ELAV4 HUMAN	P26378	ELAV like protein 4	41.74	9.24	5.56	0.601
21	SERPH HUMAN	P50454	Serpin H1	46.41	31.09	19.10	0.614
22	HNRPD HUMAN	Q14103	Heterogeneous nuclear ribonucleoprotein D0	38.41	15.10	9.42	0.624
23	BAF HUMAN	O75531	Barrier to autointegration factor	10.05	13.97	8.73	0.625
24	HNRPQ HUMAN	O60506	Heterogeneous nuclear ribonucleoprotein Q	69.56	12.95	8.18	0.631
25	PTBP1 HUMAN	P26599	Polypyrimidine tract binding protein 1	57.19	19.24	12.66	0.658
26	EF1A3 HUMAN	Q5VTE0	Putative elongation factor 1 alpha like 3	50.15	20.73	13.71	0.661
27	ROA3 HUMAN	P51991	Heterogeneous nuclear ribonucleoprotein A3	39.57	17.64	11.69	0.663
28	ROA2 HUMAN	P22626	Heterogeneous nuclear ribonucleoproteins A2 B1	37.41	72.50	48.23	0.665
29	H4 HUMAN	P62805	Histone H4	11.36	41.02	27.58	0.672
30	HNRPM HUMAN	P52272	Heterogeneous nuclear ribonucleoprotein M	77.46	8.90	6.00	0.675
31	PERI HUMAN	P41219	Peripherin	53.62	4.12	2.90	0.704
32	DHX9 HUMAN	Q08211	ATP dependent RNA helicase A	140.87	16.40	11.68	0.712
33	ILF2 HUMAN	Q12905	Interleukin enhancer binding factor 2	43.04	13.76	9.86	0.716
34	CAZA1 HUMAN	P52907	F actin capping protein subunit alpha 1	32.90	17.26	12.40	0.718
35	HNRPU HUMAN	Q00839	Heterogeneous nuclear ribonucleoprotein U	90.53	48.54	35.23	0.726

36	AINX HUMAN	Q16352	Alpha internexin	55.36	20.53	14.97	0.729
37	HNRH1 HUMAN	P31943	Heterogeneous nuclear ribonucleoprotein H	49.20	16.93	12.34	0.729
38	RLA1 HUMAN	P05386	60S acidic ribosomal protein P1	11.51	7.04	5.18	0.736
39	EF1A1 HUMAN	P68104	Elongation factor 1 alpha 1	50.11	18.99	14.05	0.740
40	HNRPK HUMAN	P61978	Heterogeneous nuclear ribonucleoprotein K	50.94	31.90	23.61	0.740
41	ROA0 HUMAN	Q13151	Heterogeneous nuclear ribonucleoprotein A0	30.82	14.77	11.04	0.748
42	HNRPR HUMAN	O43390	Heterogeneous nuclear ribonucleoprotein R	70.90	12.01	9.20	0.766
43	HNRPC HUMAN	P07910	Heterogeneous nuclear ribonucleoproteins C1 C2	33.65	68.73	52.93	0.770
44	TBB5 HUMAN	P07437	Tubulin beta chain	49.64	40.37	31.44	0.779
45	TBA1B HUMAN	P68363	Tubulin alpha 1B chain	50.12	52.71	41.18	0.781
46	NEST HUMAN	P48681	Nestin	177.33	14.42	11.28	0.782
47	HS90A HUMAN	P07900	Heat shock protein HSP 90 alpha	84.61	5.83	4.64	0.795
48	HSP7C HUMAN	P11142	Heat shock cognate 71 kDa protein	70.85	17.22	13.77	0.799
49	ILF3 HUMAN	Q12906	Interleukin enhancer binding factor 3	95.28	9.39	7.74	0.824
50	G3P HUMAN	P04406	Glyceraldehyde 3 phosphate dehydrogenase	36.03	16.66	13.85	0.831
51	MATR3 HUMAN	P43243	Matrin 3	94.56	10.88	9.46	0.869
52	VIME HUMAN	P08670	Vimentin	53.62	216.71	190.38	0.879
53	ENO1 YEAST	P00924	Enolase 1	46.79	100.00	100.00	1.000
54	TPIS HUMAN	P60174	Triosephosphate isomerase	30.77	5.83	5.92	1.015
55	RBMX HUMAN	P38159	RNA binding motif protein X chromosome	42.31	15.28	16.97	1.111

56	PROF1 HUMAN	P07737	Profilin 1	15.04	11.26	12.62	1.121
57	COF1 HUMAN	P23528	Cofilin 1	18.49	12.05	13.79	1.144
58	DESM HUMAN	P17661	Desmin	53.50	1.29	1.52	1.179
59	HS90B HUMAN	P08238	Heat shock protein HSP 90 beta	83.21	6.27	7.44	1.187
60	HNRPF HUMAN	P52597	Heterogeneous nuclear ribonucleoprotein F	45.64	4.32	6.01	1.392
61	ACTB HUMAN	P60709	Actin cytoplasmic 1	41.71	185.98	312.75	1.682
62	TBB2A HUMAN	Q13885	Tubulin beta 2A chain	49.87	2.30	3.95	1.719
63	TBB2B HUMAN	Q9BVA1	Tubulin beta 2B chain	49.92	4.43	8.74	1.972
64	ELAV2 HUMAN	Q12926	ELAV like protein 2	39.48	0.77	4.63	5.986

Supplementary Table 3. RNA sequencing analysis mapping statistics. The analysed samples were DJ-1 WT control versus DJ-1 C106DD oxidation mimic. All samples were analysed in triplicates and number of total reads, uniquely mapped reads and multi mapped reads are shown.

Sample Name	Condition	Replicate Number	Total Reads	Uniquely Mapped Reads	Multi-mapped Reads
WT1	DJ-1 WT control	1	31,542,116	27,084,153	3,807,142
WT2	DJ-1 WT control	2	27,971,043	24,062,256	3,315,992
WT3	DJ-1 WT control	3	31,306,604	26,891,059	3,739,876
DD1	DJ-1 oxidation mimic	1	17,485,020	14,830,194	2,128,032
DD2	DJ-1 oxidation mimic	2	25,494,758	21,661,059	3,251,840
DD3	DJ-1 oxidation mimic	3	23,834,874	19,990,943	3,005,557
